# Rare sugars: metabolic impacts and mechanisms of action: a scoping review

**DOI:** 10.1017/S0007114521003524

**Published:** 2022-08-14

**Authors:** Alison Smith, Amanda Avery, Rebecca Ford, Qian Yang, Aurélie Goux, Indraneil Mukherjee, David C. A. Neville, Preeti Jethwa

**Affiliations:** 1 Division of Food, Nutrition and Dietetics, School of Biosciences, University of Nottingham, Sutton Bonington Campus, Loughborough LE12 5RD, UK; 2 Mondelēz International, Nutrition Research, 91400 Saclay, France; 3 Mondelēz International, Ingredient Research, East Hanover, NJ, USA; 4 Mondelēz International, Reading Sciences Centre, Reading RG6 6LA, UK

**Keywords:** Rare sugar, d-psicose, d-tagatose, Type 2 diabetes, Obesity

## Abstract

Food manufacturers are under increasing pressure to limit the amount of free sugars in their products. Many have reformulated products to replace sucrose, glucose and fructose with alternative sweeteners, but some of these have been associated with additional health concerns. Rare sugars are ‘monosaccharides and their derivatives that hardly exist in nature’, and there is increasing evidence that they could have health benefits. This review aimed to scope the existing literature in order to identify the most commonly researched rare sugars, to ascertain their proposed health benefits, mechanisms of action and potential uses and to highlight knowledge gaps. A process of iterative database searching identified fifty-five relevant articles. The reported effects of rare sugars were noted, along with details of the research methodologies conducted. Our results indicated that the most common rare sugars investigated are d-psicose and d-tagatose, with the potential health benefits divided into three topics: glycaemic control, body composition and CVD. All the rare sugars investigated have the potential to suppress postprandial elevation of blood glucose and improve glycaemic control in both human and animal models. Some animal studies have suggested that certain rare sugars may also improve lipid profiles, alter the gut microbiome and reduce pro-inflammatory cytokine expression. The present review demonstrates that rare sugars could play a role in reducing the development of obesity, type 2 diabetes and/or CVD. However, understanding of the mechanisms by which rare sugars may exert their effects is limited, and their effectiveness when used in reformulated products is unknown.

## Graphical abstract

**Figure:**
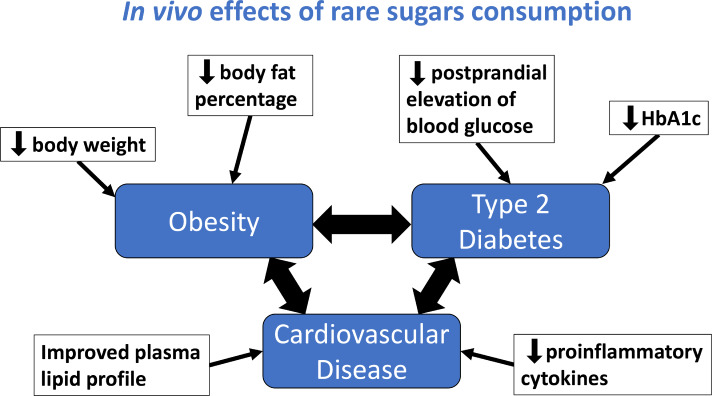

There is increasing concern over the excess intake of metabolisable free sugars, which is associated with obesity and increased risk of non-communicable diseases^(1–3)^. Many food manufacturers are reformulating products to replace sucrose, glucose or fructose with dietary fibres, polyols or high-intensity sweeteners, but alternative sweeteners may be associated with health concerns such as appetite dysregulation and glucose intolerance^(4)^. Rare sugars, defined by the International Society of Rare Sugars as ‘monosaccharides and their derivatives that hardly exist in nature’^(5)^, have attracted increasing interest as a result of recent advances in their commercial-scale biosynthesis^(6,7)^. Rare sugars are low-energy monosaccharides with similar sweetness to that of sucrose^(6)^. The rare sugars d-psicose (PSI, also known as allulose) and d-tagatose (TAG) have ‘generally recognised as safe’ status^(8,9)^. Both are already in use in products such as biscuits, chocolate, jam^(10)^, protein bars, soft drinks^(11)^ and in commercial sweetener blends^(12)^ in parts of Europe, Asia and the USA. Rare sugars have the advantage that, unlike high-intensity sweeteners, they can replace both the physical bulk and some of the sweetness of sucrose. They can therefore be used as a direct replacement for a significant portion of free sugars^(13–15)^, allowing the production of confectionery with lower energy content. PSI in particular is an attractive option for food manufacturers as it is exempted from ‘total sugars’ and ‘added sugars’ figures on nutrition labelling in the USA^(11)^.

The potential benefits of rare sugars go beyond simply replacing sucrose to reduce energy intake. Research into the potential uses of rare sugars has been ongoing since the late 1990s, primarily in East Asia, with minimal research activity in the UK. They have been shown to have a range of beneficial biological functions^(6)^, some of which could help to alleviate problems associated with the high consumption of free sugars. The biological actions of various rare sugars suggest they could contribute towards health improvements in a range of interlinked conditions, including obesity, type 2 diabetes (T2D) and CVD^(6,15,16)^. Rare sugars therefore have the potential to be used not only to replace sucrose in product reformulation but also as functional ingredients with health-promoting properties. Functional foods are foods or drinks which can have health benefits beyond their basic nutritional value^(17)^. In order for health claims to be made about a food or ingredient, there must be robust evidence that it reaches its site of action, beneficially affects a physical function or biomarker and has a direct impact on health status when consumed as part of a normal diet^(17)^. Research on rare sugars is in its early stages, and the evidence that would be required to make health claims is not yet available. More research is necessary before it can be claimed that rare sugars have health benefits in the general population.

The majority of research to date has focussed on measuring a limited range of outcomes (e.g. body composition, HbA1c levels or short-term glycaemic response) and the biological mechanisms underlying these outcomes are not yet clear. While there are several commercially available food products containing rare sugars^(10,11)^, there has been no research into the possible health benefits of these products. A detailed examination of the existing literature could help to explain mechanisms of action and highlight areas where further research is needed. Existing reviews on rare sugars have either focussed on a single sugar^(9,15,16,18,19)^ or are broad summaries of potential uses, with little focus on mechanisms of action^(6,20)^. The purpose of this scoping review is to provide an updated, comprehensive summary of the research into the potential health benefits of rare sugar consumption. The review identifies the most commonly researched rare sugars, explores their potential health benefits, mechanisms of action and possible uses and highlights gaps in the evidence. Understanding the scope of the current evidence base and its limitations is critical to improving the design and implementation of future studies.

## Methods

A scoping review differs from a systematic review, in that it aims to rapidly map the key concepts underpinning a research area. This scoping review aimed to identify primary research into the health benefits of the consumption of rare sugars, and employed the framework set out by Arksey and O’Malley^(21)^. While the objectives and methods were specified in advance, search terms and inclusion criteria were adapted during the process as the scope of the literature was identified.

### Review questions

This review seeks to answer the following questions:

1. Which rare sugars have been researched?
2. What are their known effects when consumed orally?
3. What are the mechanisms of action for these effects?
4. What are the proposed health benefits of rare sugars?
5. How might rare sugars be used to provide these health benefits?
6. What are the priorities for future research into rare sugars?

### Identifying relevant studies

In order to test possible search terms, gain an overview of the literature and define the key concepts, a limited search of the literature was performed. The search term ‘rare sugar AND (uses OR nutrition OR health)’ was used in a search of Scopus. The titles and abstracts of the articles were scanned and several recent reviews were read in full^(6,16,22)^, allowing the identification of key concepts: the most commonly researched rare sugars, and broad areas of relevant research. These concepts were used to develop a search matrix for a systematic literature search (online Supplementary Table S1) and to refine inclusion and exclusion criteria.

For the scoping review, all searches were performed in three databases (Scopus, PubMed and Web of Science) using identical search terms. The most recent searches were completed on 5 January 2021. Following title and abstract screening, the reference lists of relevant articles were searched to identify additional studies.

### Study selection

#### Inclusion and exclusion criteria

Preliminary literature searching revealed a wide range of uses for rare sugars^(6)^. While many of these are health related, some involve the use of rare sugars in an industrial, pharmaceutical or medical context. This review focuses on the health benefits of rare sugars in nutrition, therefore only includes studies where rare sugars have been administered orally *in vivo,* in humans or mammals. A significant milestone in rare sugars research was the discovery in 2004 of enzymatic methods by which rare sugars could be produced on an industrial scale^(23)^. This resulted in an increase in relevant research studies, particularly human trials. In order to rapidly identify the most relevant research, this review was therefore limited to articles published after 1 January 2004. Where relevant articles were unavailable in English, their abstracts were still included.

This review includes:

- Primary, *in vivo* research in humans or mammals in which rare sugars were administered orally.
- Studies published after 1 January 2004.

Studies were excluded if:

- The type or quantity of rare sugar was unclear (e.g. those using plant extracts)
- The rare sugar was not administered orally (e.g. solution injected or used in surgical procedures).

#### Study screening

**Fig. 1:**
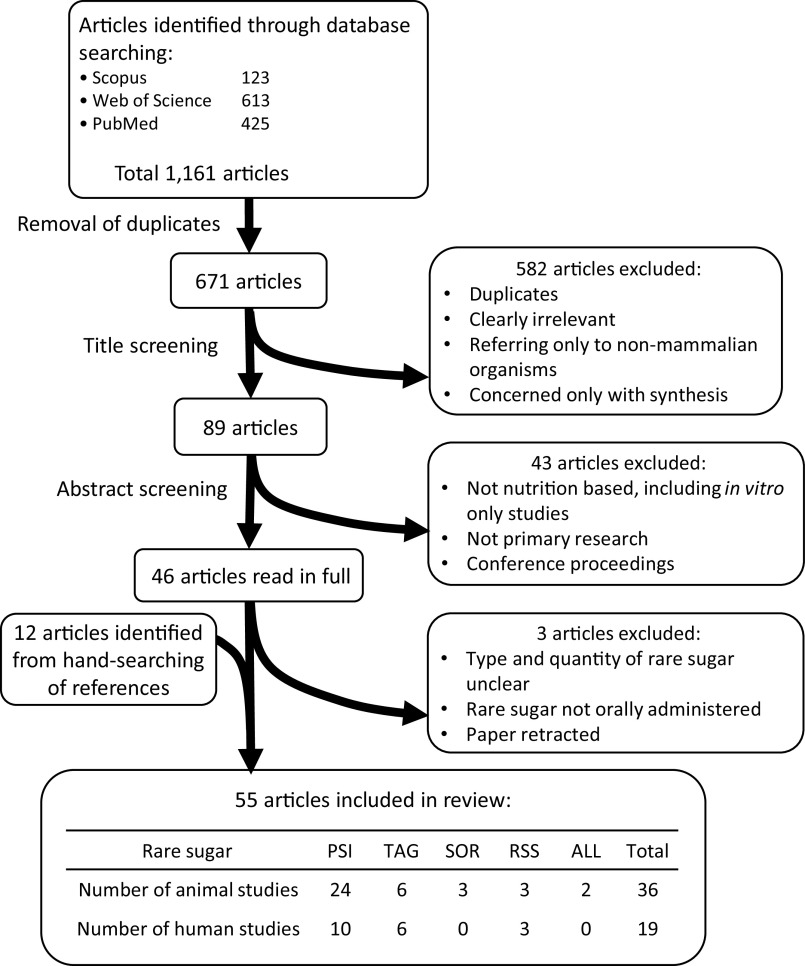
Identification and selection of relevant research. PSI: D-psicose, TAG: D-tagatose, SOR: D-sorbose, RSS: rare sugar syrup, ALL: D-allose.

The process of article screening is summarised in Fig. 1. Following database searching and removal of duplicates, title screening and abstract screening were carried out using the defined inclusion and exclusion criteria stated above.

### Charting the data

Included articles were read in full and data extracted. Microsoft Excel spreadsheets were used to allow methodical collection of available data, including the location, animal model or study population used, study design, rare sugar used, timescale, dosage, outcome measures and significant findings. Separate tables were used to record data from animal and human trials (online Supplementary Table S2a and S2b, respectively). As a scoping review aims to rapidly identify the parameters and gaps in a research area, quality of research is not a priority^(24)^; therefore, no systematic quality assurance was conducted and data from abstracts were included where full methods were not available.

### Collating, summarising and reporting the results

**Fig. 2:**
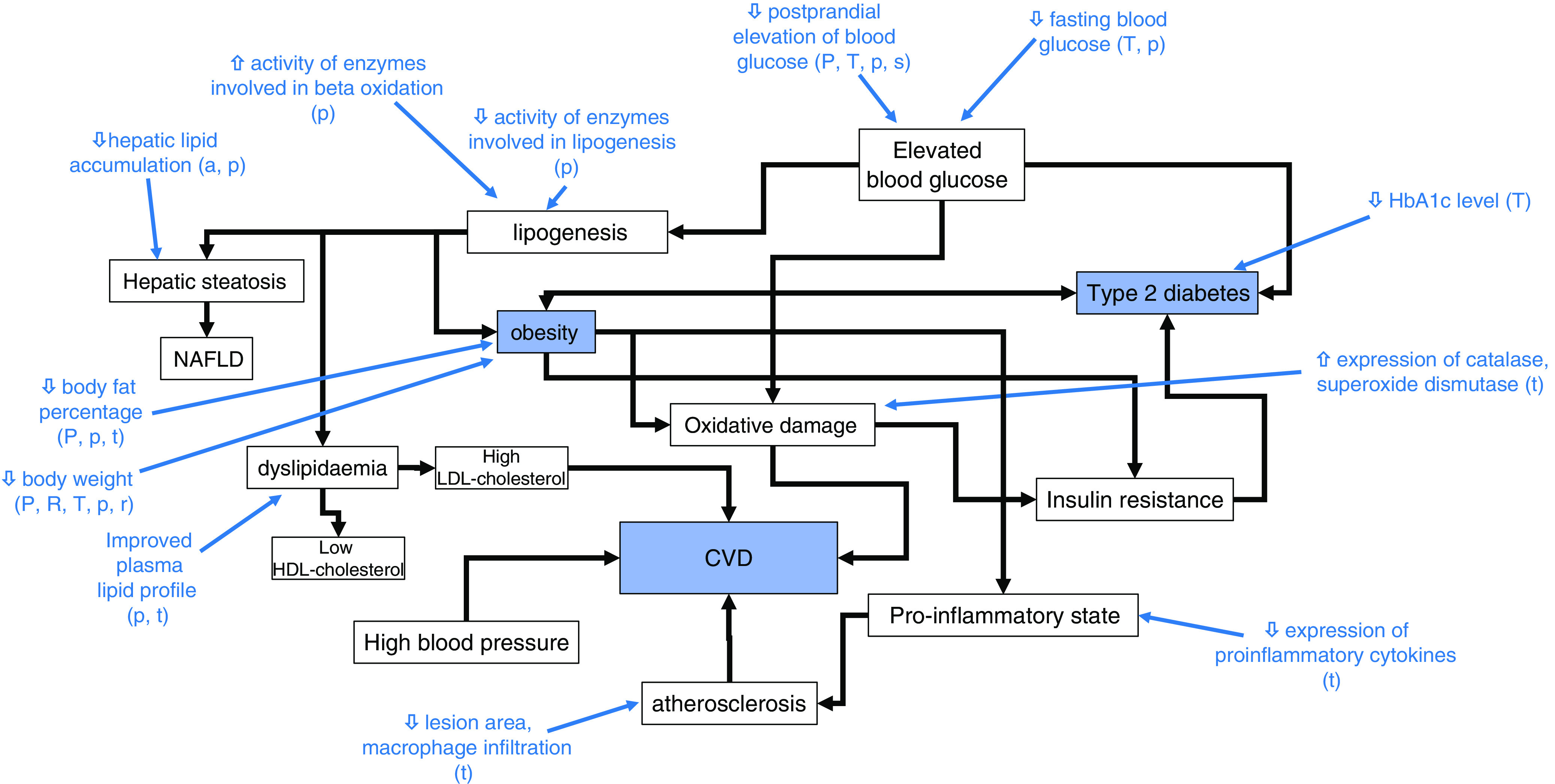
Mapping diagram to show the health benefits of rare sugars and how they are interlinked. Blue text indicates actions of rare sugars demonstrated in at least one study included in this review. Letters in brackets indicate the rare sugars involved, with capital letters denoting human studies and lower-case letters denoting animal studies: A/a – allose, P/p – psicose, S/s – sorbose, T/t – tagatose.

Study characteristics and available data were tabulated. A mapping diagram (Fig. 2) was created to summarise the proposed health benefits described in the literature and their inter-relationships. Where several studies had similar methodology, additional tables were created to enable comparison of their methods, outcomes and effect sizes.

## Results

The outcomes of literature searching and article screening are summarised in Fig. 1. A total of fifty-five articles were included in this review (see online Supplementary Tables S2a and S2b). The rare sugars identified as being most relevant from the included articles were PSI (also referred to as allulose), TAG, d-sorbose (SOR), d-allose (ALL) and rare sugar syrup (RSS, a syrup containing glucose and fructose along with around 5 % PSI and small quantities of other rare sugars, which can be economically produced by isomerisation of high fructose corn syrup under alkaline conditions). PSI, TAG and RSS are the most commonly researched rare sugars, and the only ones to have been used in human trials. There has been relatively little research into the health benefits of SOR, although some animal studies suggest it has the potential to improve glycaemic control^(25,26)^. Much of the research involving ALL uses the compound in a pharmaceutical context (injected intravenously or as an antioxidant in irrigation fluid during surgery) and, therefore, is excluded from this review. There is limited research into the use of ALL as a dietary supplement, but one study in mice indicates that it has potential to improve fatty liver disease^(27)^.

The reported *in vivo* effects of rare sugar consumption in humans included improved glycaemic control^(28–39)^, reductions in body weight^(36–39)^ and body fat^(36,39)^, and reduced LDL-cholesterol and total cholesterol^(40)^. Similar effects were reported in animal studies. Additionally, there is evidence from animal trials that rare sugar intake may also reduce hepatic lipid accumulation^(27,41–46)^, alter the gut microbiome^(44,45,47)^ and improve inflammatory^(45,47)^ and oxidative status^(48–50)^. Therefore, results on the impact of PSI, TAG, RSS, SOR and ALL on these outcomes (glycaemic control, body weight and body fat, lipid metabolism, hepatic lipid accumulation and gut microbiome) will be presented. The effect of rare sugar consumption on appetite in humans has been monitored in some studies, with inconclusive results^(51–53)^. Table 1 summarises the key effects of rare sugar consumption as reported in the studies included in this review^(54)^. Taken as a whole, the evidence suggests that rare sugars may have the potential to improve or reduce the risk of obesity, T2D, CVD and fatty liver disease. The mapping diagram shown in Fig. 2 summarises how these interlinked conditions could be affected by rare sugar consumption. Importantly, the diagram highlights that the effect of lowering postprandial glucose levels may lead to multiple health benefits. However, the majority of the evidence to date is from animal studies, and the mechanisms of action of the rare sugars are not understood. The extent to which rare sugars can affect pathways that lead to the alleviating of disease states is unclear.

**Table 1.** Summary of the reported health benefits of rare sugar consumption (including only studies reporting significant results)§

Observed effect of rare sugar	Human studies				Animal studies			
	Subjects with T2D, hyperglycaemia or obesity		Healthy subjects		Animal models of metabolic disease		Normal animals	
Reduced PEBG	PSI	Hayashi 2010^(28)^	PSI	Matsuo 2011^(30)^ *,†	PSI	Hossain 2011^(43)^, 2012^(59)^,	SOR	Oku 2014^(25)^
		Noronha 2018^(29)^		Yamada 2018^(31)^ *,†		Iwasaki 2018^(51)^		
	TAG	Kwak 2013^(33)^		Iida 2008^(32)^ †		Pongkan 2020^(64)^	RSS	Shintani 2017^(74)^
			RSS	Nakamura 2017^(34)^ *,†				
Improved long-term glycaemic control	TAG	Ensor 2014^(38)^, 2015^(40)^			PSI	Baek 2010^(58)^	PSI	Iida 2013^(66)^ ‡
						Do 2019^(41)^ ‡		
						Han 2016^(42)^		
						Hossain 2011^(43)^. 2012^(59)^. 2015^(60)^		
Reduced body weight	PSI	Han 2018^(36)^	RSS	Hayashi 2014^(39)^ ‡	PSI	Han 2016^(42)^ ‡, 2020^(44)^ ‡, 2020^(45)^ ‡	PSI	Huang 2018^(76)^ †
		Donner 2010^(37)^				Baek 2010^(58)^		Nagata 2015^(65)^ *,‡
	TAG	Ensor 2014^(38)^				Choi 2018^(71)^ *,‡		Yagi 2009^(54)^
						Chung 2012^(72)^	RSS	Iida 2013^(66)^ ‡
						Do 2019^(41)^ ‡		Shintani 2017^(74)^
						Hossain 2011^(43)^, 2012^(59)^	ALL	Iga 2010^(67)^
						Itoh 2015^(46)^		
						Kim 2017^(73)^ ‡		
						Ochiai 2013^(62)^		
						Hossain 2015^(60)^		
					TAG	Williams 2015^(91)^ *,‡		
Reduced body fat	PSI	Han 2018^(36)^	RSS	Hayashi 2014^(39)^ ‡	PSI	Han 2020^(44)^ ‡	PSI	Chen 2017^(48)^ *,‡,†, Chen 2019^(49)^ *,‡
						Choi 2018^(71)^ *,‡		
						Hossain 2011^(43)^		
						Itoh 2015^(46)^		
						Kim 2017^(73)^ ‡		
						Ochiai 2013^(62)^		
					RSS	Ochiai 2017^(63)^ *,‡		
Improved plasma lipid profile	TAG	Ensor 2015^(40)^			PSI	Han 2016^(42)^ ‡, 2020^(44)^ ‡, 2020^(45)^ ‡	PSI	Chen 2017^(48)^ *,‡,†, Chen 2019^(49)^ *,‡
						Baek 2010^(58)^		
						Choi 2018^(71)^ *,‡		
						Do 2019^(41)^ ‡		
						Ochiai 2013^(62)^		
						Hossain 2015^(60)^		
						Kim 2017^(73)^ ‡		
						Kanasaki 2019^(61)^ *,‡		
					TAG	Williams 2015^(91)^ *,‡		
Reduced hepatic lipid accumulation					PSI	Han 2016^(42)^ ‡, 2020^(44)^ ‡, 2020^(45)^ ‡	PSI	Chen 2019^(49)^ *,‡
						Do 2019^(41)^ ‡		
						Hossain 2011^(43)^		
						Itoh 2015^(46)^		
					ALL	Yamamoto 2017^(27)^		
Improved gut microbiome					PSI	Han 2020^(44)^ ‡, 2020^(45)^ ‡		
					TAG	Son 2019^(47)^		
Reduced inflammation					PSI	Han 2020^(45)^ ‡		
					TAG	Son 2019^(47)^		
Improved oxidative status					PSI	Pratchayasakul 2020^(50)^ †	PSI	Chen 2017^(48)^ *,‡,†, Chen 2019^(49)^ *,‡

PEBG, postprandial elevation of blood glucose.

* Indicates studies in which there was a possible energy deficit in the experimental group.

‡ Indicates studies in which the rare sugar replaced another carbohydrate in the experimental diet.

† Indicates studies available as abstracts only.

§ Studies reporting inconclusive or non-significant results have not been included.

### Rare sugars and glycaemic control

There is evidence from human trials that both PSI and TAG, when consumed with a carbohydrate load, can reduce the resulting elevation in blood glucose in people with hyperglycaemia^(28,29,33)^ (Table 2). Most of these studies involved a control group consuming the same carbohydrate load, so the effect can be attributed to the rare sugar rather than a simple decrease in carbohydrate intake. It should be noted that the reduction in the incremental AUC for glucose was relatively small (4–11 % with PSI, 4 % with TAG) compared with the effects of oral hypoglycaemic agents^(55)^.

**Table 2.** Summary of human trials examining the effect of rare sugars on postprandial blood glucose elevation*

Study and location	Study population	Trial design	RS dose	CHO load	Ratio RS:CHO	Control	Difference in AUC	*P*	Conclusions
Braunstein *et al.*, 2018^(56)^, Canada	Healthy volunteers (*n* 25). Age 37 (sd 16). BMI 24·7 (sd 3·4)	Randomised, controlled, double-blind, multiple-crossover	5 g PSI in glucose solution	75 g glucose solution	1:15	No addition to CHO load	–35 (sd 22) mmol/l × min (15·6 % reduction)	0·11	No significant effect on plasma glucose iAUC compared with 0 g PSI control
			10 g PSI in glucose solution		2:15		–23 (sd 22) mmol/l × min (10·3 % reduction)	0·07	
Hayashi *et al.*, 2010^(28)^, Japan	Borderline diabetes (*n* 15) and healthy volunteers (*n* 11). Age 55·0 (sd 11·4). BMI 24·9 (sd 4·4)	Randomised, controlled, double-blind, crossover	5 g PSI single dose in tea given with meal	Standard meal (425 kcal, 84·5 g CHO, 13·3 g protein, 3·7 g fat)	1:17	Aspartame	–743·3 mg × min/dl overall for meal (11·5 % reduction)	< 0·01	AUC for PSI meal was significantly less than control aspartame meal overall and in subgroup of subjects with borderline diabetes but not in subgroup of healthy participants
Kimura et al, 2017^(57)^, Japan	Healthy volunteers (*n* 13). Age 35·7 (sd 2·0). BMI 20·9 (sd 0·7)	Randomised, controlled, single-blind, crossover	5 g PSI single dose in solution, 30 min before meal	Standard meal (571 kcal; 61 % of energy as CHO, 25 % as fat, 14 % as protein. (estimated 93 g CHO)	1:19	Aspartame	NA	NA	PSI supplementation gave significantly lower change in plasma glucose at 90 min only, compared with aspartame control. No significant difference in plasma insulin
Noronha *et al.*, 2018^(29)^, Canada	Subjects with T2D, controlled with diet or OHA, not insulin (*n* 24). Age 66 (sd 1·2). BMI 27·0 (sd 0·9)	Randomized controlled, double-blind, crossover	5 g PSI in glucose solution	75 g glucose solution	1:15	No addition to CHO load	–48·1 mol × min/l (6·2 % reduction)	0·051	Significant linear dose–response gradient for reduction in AUC for glucose
			10 g PSI in glucose solution		2:15		–60·1 mol × min/l (7·7 % reduction)	0·015	
Matsuo & Lu, 2011^(30)^, Japan†	Healthy volunteers (*n* 44)	No crossover, no info on randomisation or blinding	6 g PSI single dose before meal	Normal lunch selected by subjects (636 kcal, 87·6 g CHO for males, 513 kcal, 18·9 g CHO for females)	1:15 male, 1:3 female	6 g d-fructose	NA	NA	Postprandial glycaemic response significantly lower after PSI compared with D-fructose control
Iida *et al.*, 2008^(32)^, Japan†	Healthy volunteers (*n* 20)	Randomised, single-blind crossover	2·5, 5 or 7·5 g PSI	75 g maltodextrin solution	1:10, 1:15 or 1:30	No addition to CHO load	NA	NA	Dose-dependent reduction in postprandial blood glucose, with significant effects at doses of 5 g or greater
Tanaka *et al.*, 2020^(35)^ Japan†	Healthy volunteers (*n* ?)	Randomised, single blind crossover	0, 1·8, 3·6 or 12·5 g PSI in 50 g chocolate	50 g chocolate (carbohydrate content not provided)	NA	NA	NA	NA	Reduction in postprandial blood glucose and insulin with PSI compared with control
Nakamura *et al.*, 2017^(34)^, Japan†	Healthy volunteer (*n* 10)	Randomised, single-blind, placebo-controlled crossover	0, 15, 25 or 35 g RSS	Total 50 g CHO-sucrose with part replaced by RSS	3:7, 5:5 or 7:3	Sucrose	NA	NA	Compared with 100 % sucrose control, 5:5 and 7:3 ratios gave significant reduction in iAUC for glucose and insulin. 3:7 ratio gave significant reduction in iAUC for insulin but not glucose
	Healthy volunteers (*n* 12)		5 g RSS	Total 10 g CHO-sucrose with half replaced by RSS	1:1	Sucrose			Significant reduction in iAUC for glucose and insulin with 1:1 RSS:sucrose compared with sucrose alone
Yamada *et al.*, 2018^(31)^, Japan†	Healthy volunteers (*n* 14)	Randomised, single blind, placebo-controlled crossover	Half sucrose replaced with RSS	Sucrose (no info on amount)	1:1	Sucrose	NA	NA	Significant reduction in iAUC for glucose compared with sucrose control
	Healthy volunteers (*n* 10)		Sucrose replaced with RSS		3:10 and 5:5	Sucrose			Significant reduction in iAUC for glucose compared with sucrose control
Kwak *et al.*, 2013^(33)^, Korea	Healthy volunteers (*n* 52). Age 35·8 (sd 1·45). BMI 23·7 (sd 0·54)	Randomised, double-blind, placebo-controlled crossover	5 or 10 g TAG in drink before a meal	Standard meal, 356 kcal of which 60 % (53 g) CHO	1:10 or 1:5	Sucralose-erythritol drink	–3·3 mg/dl per h (1·32 % reduction)	NS	Significant reduction in iAUC only in hyperglycaemic subjects
	Hyperglycaemic subjects (impaired fasting glucose or newly diagnosed T2D, *n* 33). Age 57·2 (sd 1·71). BMI 25·0 (sd 0·46)						–15·4 mg/dl per h (4·0 % reduction)	< 0·05	
Wu *et al.*, 2012^(52)^, Australia	Healthy volunteers (*n* 10). Age 28·2 (sd 4·0). BMI 25·5 (sd 1·5)	Randomised, single-blind, placebo-controlled crossover	40 g TAG–isomalt mixture (16 g TAG), 20 min before meal	Standard meal containing 63 g CHO	1:4	Sucralose preload	+0·5 mmol/l × min (0·25 % increase)	NS	Significant increase in iAUC with glucose preload, but no significant differences between TAG and control

RS, rare sugar; PSI, d-psicose; CHO, carbohydrate; T2D, type 2 diabetes; OHA, oral hypoglycaemic agents; NA, not available; RSS, rare sugar syrup; TAG, d-tagatose; NS, not significant.

* Difference in iAUC (incremental area under the curve) for glucose is the difference between rare sugar treatment group *v.* control group in 120 min following ingestion of carbohydrate load. *P*-values are for significance of difference as stated in the referenced article. Ages are given in years, and BMI is given in kg/m^2^. †Articles not available in English

In studies where PSI^(28,56,57)^ or TAG^(33,52)^ was consumed by healthy volunteers, no significant reductions in the incremental AUC for glucose were observed, although Kimura *et al.* ^(57)^ reported significantly lower blood glucose at 90 min after PSI was consumed before a standard meal. Some studies did report significant reductions in the postprandial elevation of blood glucose (PEBG) with PSI^(30)^ or RSS^(31,34)^ consumption in healthy volunteers, but in these studies the experimental groups consumed reduced carbohydrate loads compared with the control groups. The effects of SOR and ALL on glycaemic response have not been studied in humans, but one study in Wistar rats^(25)^ reported a reduction in peak blood glucose concentration when SOR was given alongside a sucrose load.

The effect of longer-term rare sugar consumption on glycaemic control has also been investigated, with inconsistent results. Three studies examined the long-term effects of TAG in subjects with T2D^(37,38,40)^. Of these, two found small but significant decreases in HbA1c after 12 months of regular TAG consumption^(38,40)^. One study^(36)^ investigated the effect of longer-term PSI consumption on glycaemic control in overweight individuals and found no significant change in fasting blood glucose or HbA1c after 12 weeks.

The animal studies included in this review highlight the different effects of rare sugars on long-term glycaemic control in different animal models. Four studies investigated the effect of PSI consumption in animal models of the metabolic syndrome (*db/db* mice^(58)^ or Otsuka Long-Evans Tokushima Fatty (OLETF) rats^(43,59,60)^), and all found significantly reduced plasma glucose with PSI compared with the control groups. A further six studies induced obesity and hyperglycaemia in wild-type animals by feeding high-sucrose or high-fat diets. Of these, three found no significant differences in blood glucose or insulin with PSI^(61,62)^ or RSS^(63)^ feeding. Reductions in fasting blood glucose were reported in two studies feeding PSI to mice with diet-induced obesity (DIO)^(41,42)^, and Pongkan *et al.* ^(64)^ found significant reductions in fasting insulin levels and insulin resistance when PSI was fed to obese Wistar rats. Of the four studies in which there was no metabolic disorder, two reported a reduction in insulin levels with PSI^(65)^ or RSS^(66)^, with Iida *et al.* also reporting reduced fasting blood glucose^(66)^. The included animal studies using ALL^(27,67)^, SOR^(26,68)^ and TAG^(68–70)^ found no significant difference in blood glucose, although Yamada *et al.* ^(26)^ reported a reduction in non-fasting serum insulin levels after 4 weeks of SOR feeding.

### The effect of rare sugar consumption on body weight and body fat

Of the twenty-seven animal studies where rare sugars were fed as part of the diet (typically 2–5 % for PSI, RSS, ALL or SOR, 30 % for TAG) or in drinking water (1–2 % solution) for periods of 4 weeks or more, twenty-two studies found significant reductions in body weight with rare sugar consumption (see Table 1). In eighteen of the studies, where adipose tissue mass was an outcome measure, significant reductions were reported in seventeen studies^(41–46,48,49,59,62,63,66,71–74)^. Many of these studies were designed to reduce or eliminate the effect of differences in energetic intake, either using a paired-feeding approach or feeding isoenergetic diets and carefully monitoring feed intake, but in some cases there was an energy deficit in rare-sugar-fed animals.

Although few long-term clinical trials have been conducted, two trials in healthy adults found reductions in BMI and body fat percentage (BFP) when drinks containing PSI^(36)^ or RSS^(39)^ were consumed regularly over 12 weeks. Han *et al.* ^(36)^ reported modest but significant reductions in BMI (–0·38 kg/m^2^) and BFP (–0·74 %) in subjects consuming 14 g PSI per day, with significant differences compared with a sucralose control group in which these parameters were unchanged. Similarly, Hayashi *et al.* ^(39)^ found significant reductions in body weight (–1·85 kg), BMI (–0·68 kg/m^2^) and BFP (–1·72 %) in subjects consuming 30 g RSS per day, while no significant changes in these parameters were seen in control groups consuming isoenergetic drinks containing 28 g high fructose corn syrup. In each of these studies, food intake was recorded using 24 h recalls^(36)^ or 3-d food diaries^(39)^, and no significant differences between groups were reported. Of three studies^(37,38,40)^ where TAG was given regularly to adults with T2D, two found significant decreases in body weight from baseline, although neither of these studies had a control group^(37,38)^. A large phase 3 clinical trial using the same dosing regimen (15 g TAG three times daily before meals) found no significant differences in body weight between the TAG group and the control group who consumed a sucralose placebo^(40)^. None of the clinical trials using TAG reported food intake during the treatment period, so the potential contribution of energy reduction and the effect of TAG on appetite are not known.

Some of the animal studies in this review reported that PSI consumption resulted in decreased food intake^(43,51,60,66)^, indicating a potential effect of PSI on appetite, although in most of the animal studies there was no significant difference in food intake.

Only one of the clinical trials in this review reported on differences in appetite with rare sugar consumption, and this was in the context of a study of gastrointestinal tolerance. Participants were given gradually increasing doses of 0·2–1 g PSI per kg body weight, with gradually increasing daily frequency, over 1 week to find the maximum daily dose for regular ingestion. Diminished appetite, as one of a range of reported adverse effects, was self-reported by two of the nineteen participants on day 8, after consuming the highest dose of 1 g PSI per kg body weight^(75)^.

### Rare sugar consumption and lipid metabolism

Research methodologies used in animal studies include the measurement of plasma, hepatic and faecal triglyceride, cholesterol and NEFA, as well as the expression and activities of enzymes involved in lipid metabolism. Of the twenty-five studies where blood lipids were measured, seventeen used animal models of obesity (leptin deficient *ob/ob* mice or animals with DIO). The reported effects of rare sugar consumption on lipid metabolism are contradictory: of all the animal studies measuring plasma lipids, only thirteen found overall beneficial effects of rare sugar consumption (reduction in plasma triglyceride or total cholesterol, or increased ratio of HDL- to LDL-cholesterol). Two of these were in Wistar rats without obesity^(49,76)^, although in both of these studies the diet of the PSI-fed rats was lower in energy than that of the control group. Nine studies reported reduced LDL-cholesterol or non-HDL-cholesterol with PSI consumption^(42,44,45,49,60–62,71,76)^, and in four of these studies the HDL:LDL-cholesterol ratio was increased^(42,45,61,71)^. However three studies found no significant effects of PSI on plasma cholesterol^(41,58,64)^, while two reported increased plasma total cholesterol and LDL-cholesterol with PSI^(72)^ or TAG^(70)^ administration. It should be noted that all but one of these studies were carried out in rat or mouse models, in which cholesterol metabolism differs significantly from that of humans^(61)^. Kanasaki *et al.* ^(61)^ conducted a study in which PSI was fed to Syrian hamsters as part of a high fat diet over 8 weeks and found no significant differences in plasma total cholesterol, although the HDL:LDL-cholesterol ratio was increased.

There is more consensus in the reported effects of rare sugars on lipid metabolism enzyme activity. In general, PSI consumption tends to increase the activity of enzymes involved in *β*-oxidation of lipids and decrease the activity of enzymes involved in lipogenesis (Table 3). For example, Do *et al.* ^(41)^ fed isoenergetic high fat diets with or without 5 % PSI supplementation to mice for 8 weeks and found that the livers of PSI-fed mice had reduced activity of phosphatidate phosphatase and glucose-6-phosphate dehydrogenase and increased activity of carnitine palmitoyltransferase 1. In several studies, the observed changes in enzyme activity were accompanied by reductions in adipose tissue weight^(41,48,71,73)^, although in two shorter studies the reductions did not reach significance^(65,68)^. Interestingly, when Nagata *et al.* ^(68)^ compared the effects of 3 % PSI, TAG and SOR in the diets of rats, they found that lipid metabolism enzymes were affected differently by the different rare sugars, for example, the activity of fatty acid synthase was decreased in PSI-fed rats but increased in the TAG-fed group.

**Table 3.** The effects of *in vivo* PSI administration on the enzymes involved in lipid metabolism

Enzyme	Role	Effect of PSI	References
Hepatic lipase	Hydrolysis of triacylglyceride	Increased activity	^(48)^
Hepatic CPT1	Catalyses the rate-limiting step in the *β*-oxidation of long-chain fatty acids	Increased expression or activity	^(41,71,73)^
Hepatic ME	Catalyses conversion of malate to pyruvate, replenishing TCA cycle intermediates. Provides a source of NADPH for lipogenesis	Decreased activity	^(71)^
Hepatic G6PDH	Provides a source of NADPH for lipogenesis	Decreased activity	^(65,68,71,73)^
ACC	Catalyses the committed step in fatty acid synthesis	Reduced expression	^(48)^
FAS	Catalyses the synthesis of long-chain fatty acids	No significant difference in activity	^(41)^
		Decreased activity or expression	^(41,48,68,71,76)^
Adipose tissue CPT1	Catalyses the rate-limiting step in the *β*-oxidation of long-chain fatty acids	Increased expression	^(71)^
HSL	Hydrolysis of long-chain fatty acids inhibited by insulin	Increased expression	^(41,48)^
PAP	Catalyses the conversion of phosphatidate to diacylglycerol, regulates TAG synthesis	Decreased activity	^(68,73)^
LPL	Hydrolyses TAG in lipoproteins	Decreased expression	^(41)^
ACAT	Catalyses key step in the mevalonate pathway, promotes cholesterol storage	Decreased activity	^(73)^
PCSK9	Binds to LDL receptor, reducing LDL-R recycling	Lower serum level	^(61)^
HMGCR	Catalyses the rate-limiting step in cholesterol synthesis	Increased activity	^(73)^

CPT, carnitine palmitoyltransferase; ME, malic enzyme; G6PDH, glucose 6-phosphate dehydrogenase; ACC, acetyl-CoA carboxylase; FAS, fatty acid synthase; HSL, hormone-sensitive lipase; PAP, phosphatidate phosphatase; LPL, lipoprotein lipase; ACAT, acetyl-co-enzyme A acetyltransferase; PCSK9, proprotein convertase subtilisin/kexin type 9; HMGCR, 3-hydroxy-3-methyl-glutaryl-co-enzyme A reductase.

### Rare sugars and hepatic lipid accumulation

Although there are conflicting results concerning the effects of rare sugars on hepatic triglyceride and cholesterol content, there appears to be a consistent protective effect against hepatic lipid accumulation with rare sugar consumption. All eight of the animal studies in which hepatic lipid was measured found that rare sugar consumption dramatically reduced lipid accumulation. In these studies, PSI^(41–46,49)^ or ALL^(27)^ was fed to genetically obese or DIO animals and, while obese control groups developed hepatic fibrosis or ballooning degeneration, the livers of PSI-fed animals were found to be similar to non-obese controls^(43–46)^.

### Rare sugars and the gut microbiome

Two recent papers by Han *et al.* ^(44,45)^ explored the effects of PSI consumption on the gut microbiome, as a possible mechanism for its observed anti-diabetic effects. In these studies, PSI was fed as 5 % of a high-fat diet to mice, with control groups pair fed isoenergetic high-fat diets. As well as reduced adipose tissue mass, serum lipids and hepatic lipids, these studies reported improved microbiome diversity; the microbiota of mice fed PSI with a high-fat diet was similar to that of mice fed a normal diet. TAG, too, has been found to be beneficial for gut microbiota in mice with induced colitis: in a study by Son *et al.* ^(47)^, TAG (25 mg), *Lactobacillus rhamnosus* (109cfu) or a combination of the two treatments were administered every other day by oral gavage, with a control group given saline. Symptoms of colitis were reduced in TAG-fed groups, and synergistic effects were observed when TAG was fed alongside probiotics. There were significant reductions in the pro-inflammatory cytokines IL-6 and IL-10 with both TAG and *Lactobacillus* alone, and additionally a reduction in TNF*α* with combination treatment.

## Discussion

This scoping review found that there is evidence, primarily from animal trials, for beneficial effects of dietary PSI consumption, particularly anti-hyperglycaemic and hypolipidaemic effects. PSI, therefore, could be a useful alternative for free sugars and assist with prevention strategies for obesity and T2D. However, evidence from human trials is limited and research gaps remain. The actions of other rare sugars are less well-researched, but TAG has potential beneficial effects in the regulation of blood glucose. The mapping diagram (Fig. 2) illustrates how the known actions of rare sugars could contribute to important health benefits linked to obesity, T2D and CVD. The majority of the studies reporting beneficial effects have involved animal models of metabolic disorders, or human subjects with hyperglycaemia, obesity or T2D (see Table 1). The potential health benefits of rare sugars as part of an ongoing normal diet in healthy individuals are unclear.

### Unpacking the mechanisms of action of rare sugars

As outlined in Fig. 2, the observed *in vivo* effects of rare sugars are extensively interlinked. The reported effects of rare sugar intake could provide health benefits related to obesity, T2D, CVD and non-alcoholic fatty liver disease (NAFLD), but the precise mechanisms by which rare sugars exert their effects are not yet understood. Potential mechanisms of action include improvements in glycaemic control, altered lipid metabolism, reduced appetite, reduced inflammation and improvements in the gut microbiome. These factors will be discussed in the following sections.

The wide range of study types and methods used in researching rare sugars has resulted in some gaps in our understanding of their mechanisms of action, for example, where one sugar has been shown to influence an outcome, biomarker, pathway or gene, which may not have been investigated using other rare sugars. An understanding of the mechanisms of action is important when considering effects in different populations, and possible synergistic effects *in vivo.*

#### Glycaemic control: alteration in carbohydrate absorption and metabolism

As summarised in Table 2, PSI and TAG have both been shown to reduce the elevation in blood glucose when given before or alongside a carbohydrate load. There have been several suggested mechanisms for this effect, including reduced digestion and absorption of dietary carbohydrates, enhanced glucose uptake from the plasma and stimulation of insulin secretion.

SOR and TAG have both been found to inhibit sucrase and maltase enzymes from rat intestines^(25)^, suggesting that reduced breakdown of disaccharides could be a mechanism by which rare sugars suppress PEBG. However, it does not fully account for the reduction in PEBG when rare sugars are given with a carbohydrate load composed entirely of glucose, as observed by Noronha *et al.* ^(29)^ and in several animal studies^(43,58–60)^. It is clear that other mechanisms of action also play a part.

Around 70 % of ingested PSI is absorbed in the small intestine^(60)^. While glucose is transported largely by the sodium-glucose linked transporter-1, both fructose and PSI enter enterocytes via the GLUT5. Efflux from enterocytes for all three monosaccharides involves the GLUT2 transporter^(77)^. This raises the possibility that PSI could reduce the absorption of both glucose and fructose by competition for sugar transporters. Indeed, TAG has been shown to reduce fructose absorption by 26 % over 60 min when administered to rats alongside equal quantities of fructose^(78)^. It would be useful to determine the transport pathways of each rare sugar, and the extent to which they can slow the transport of fructose and glucose.

A further potential mechanism for the suppression of PEBG is through enhanced glucokinase (GK) translocation. GK catalyses the first step in the metabolism of glucose for the synthesis of glycogen and triacylglycerides and is, therefore, critical in hepatic glucose metabolism. It is regulated by transcriptional changes and by translocation from the nucleus to the cytoplasm in the fed state^(79)^. This translocation of GK has been shown to be lower in hyperglycaemic or diabetic animal models, such as OLETF rats^(59)^. The translocation of GK was enhanced in both OLETF rats^(43)^ and non-diabetic Wistar rats^(74)^ fed PSI. An increase in translocation of GK to the cytoplasm increases hepatic glucose uptake and contributes to better short-term regulation of blood glucose^(79)^.

It is possible that the reduced PEBG observed with rare sugar administration is related to increased insulin secretion, stimulated by incretin hormones. These hormones, for example, glucagon-like peptide 1 (GLP-1) and gastric inhibitory peptide, are released in response to the presence of nutrients in the duodenum and enhance the glucose-stimulated release of insulin from the pancreatic islets. One study in mice found that oral PSI administration stimulated GLP-1 release, leading to increased plasma insulin and reduced plasma glucose after intraperitoneal glucose injection^(51)^. Evidence from human trials, however, does not support this mechanism of action. One study in healthy volunteers reported that TAG stimulated GLP-1 release, but this did not lead to significant differences in blood glucose or insulin after a meal^(52)^. Additionally, several studies in humans have demonstrated that PSI^(29,57)^, TAG^(33)^ or RSS^(31,34)^ consumption can reduce the incremental AUC for glucose following a carbohydrate load, but with no significant effect on plasma insulin. None of these trials reported measurements of incretin hormone levels. Insulin and incretin hormones play a vital role in glycaemic control, and the effect of rare sugar intake on insulin and incretin release in humans requires further investigation.

The relative contribution of these mechanisms *in vivo* is unknown and may be different for different rare sugars. Postprandial blood glucose shows high inter-individual variation^(56)^ and is affected by the action of insulin, glucagon and gut peptides, so the efficacy of rare sugars in suppressing PEBG is likely to vary between different animal species or human study participants. Future studies should aim to recruit sufficient participants to overcome the effects of inter-individual variation and should measure not only blood glucose but also insulin and incretin hormone levels following the ingestion of rare sugars. Such studies should also trial the intake of rare sugars in food products, similar to those currently marketed, to ascertain whether significant differences in postprandial glycaemic response are observed compared with a standard product.

While regular TAG consumption has been shown to reduce HbA1c^(38,40)^, regular PSI consumption showed no significant effects on glycaemic control^(36)^. The factors affecting long-term glycaemic control are complex, and research in humans is complicated by changes in treatment regimens in subjects with T2D^(38)^. The HbA1c measurement commonly used in diabetes management reflects average plasma glucose over the previous 8–12 weeks, but does not take into account glycaemic variation during that time^(80)^. PSI and TAG can both reduce PEBG (see Table 2), so it is possible that dietary PSI or TAG could reduce damaging episodes of both hypo- and hyperglycaemia without significantly reducing HbA1c measurements. Further long-term, large-scale studies are necessary to evaluate this, potentially using markers of short-term glycaemic control such as 1,5-anhydroglucitol^(81)^.

#### Alterations in lipid metabolism

One consistently observed *in vivo* effect of rare sugar consumption, in both animals and humans, is a reduction in BFP and adipose tissue weight^(36,39,41–46,48,49,59,62,63,66,71–74)^. Several animal studies have also reported a protective effect of rare sugars against the hepatic steatosis that results from a high-fat diet^(27,41–43,45,46,49,82)^. The effects of rare sugars on serum and liver lipids are less consistent, and the precise mechanisms for the hypolipidaemic effects of rare sugars are not well understood.

As shown in Table 3, the intake of rare sugars appears to reduce the activity of lipogenic enzymes and increase the activity of those involved in *β*-oxidation. These changes are a plausible mechanism for the reduction in adipose tissue mass or BFP observed with regular rare sugar intake. Additionally, several studies observed significant reductions in the activity of enzymes involved in lipogenesis in the liver as a result of PSI feeding (Table 3). These changes, as well as contributing to reductions in hepatic lipid accumulation, could also affect plasma lipid profiles. An increase in the activity of hepatic lipase in serum and liver, for example, could contribute to the reduction in plasma triglyceride observed in several studies^(41,42,48,71)^.

**Fig. 3:**
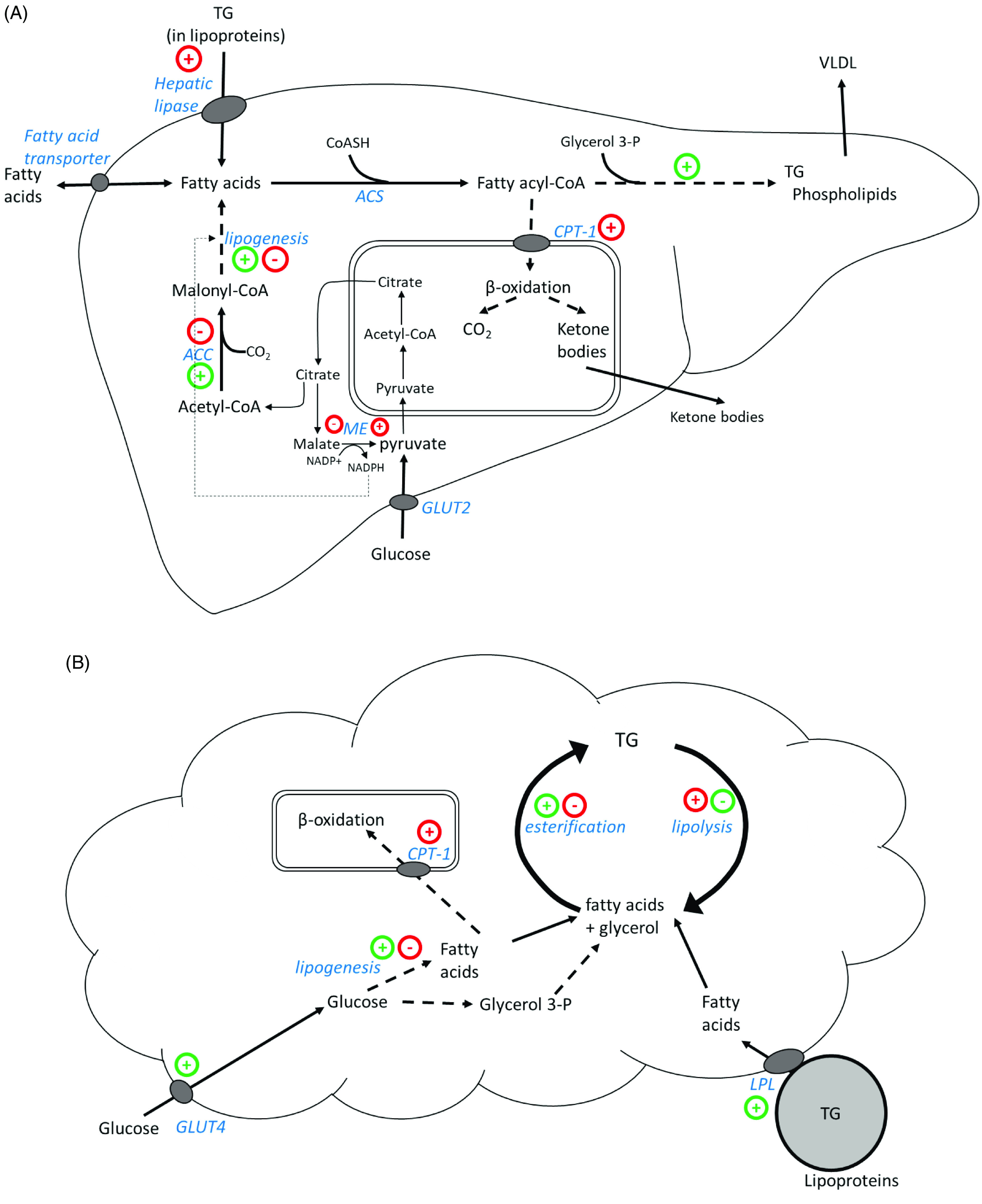
Outline of fatty acid metabolism in the liver (A) and adipose tissue (B), highlighting the effects of insulin and PSI. Green (+) or (-) indicates increased or decreased expression or activity stimulated by insulin. Red (+) or (-) indicates increased or decreased expression or activity as a result of PSI consumption. TG: triacylglycerol, VLDL: very low density lipoprotein, ACS: acyl-CoA synthase, ACC: acetyl-CoA carboxylase, CPT-1: carnitine-palmitoyl transferase 1, ME: malic enzyme, GLUT2: glucose transporter 2, GLUT4: glucose transporter 4, LPL: lipoprotein lipase. Diagrams adapted from Frayn, 2019^(83)^, p131 & 133.

Lipid metabolism is affected by blood glucose concentration, via both insulin-dependent and insulin-independent pathways^(83)^. Figure 3 outlines some of the pathways of fatty acid metabolism in the liver and adipose tissue, showing potential mechanisms by which PSI could reduce lipid accumulation. Importantly, PSI appears to oppose the effect of insulin on several enzymes and pathways involved in lipid metabolism. A key question to be addressed is whether the observed changes in enzyme activity, and the resulting reduction in lipid accumulation, are direct effects of rare sugars or a result of changes in blood glucose and insulin.

The accumulation of lipid in non-adipose tissue is considered to be a factor in several non-communicable diseases. Fat infiltration in liver and muscle tissue is associated with insulin resistance^(84)^, and in NAFLD lipid accumulates in hepatocytes causing liver damage^(85)^. NAFLD is closely associated with insulin resistance and obesity and is one of the most common causes of chronic liver disease worldwide, with estimated global prevalence of 24 %^(86)^. While rare sugars have been found to protect against lipid accumulation in the liver in DIO animals, the effect of rare sugars on lipid accumulation in muscle tissue does not appear to have been studied. Research in this area could provide useful insights into the potential therapeutic benefits of rare sugars.

#### Alterations in incretin response and appetite regulation

The question of whether rare sugars can affect appetite in people has not been extensively researched, and most of the animal studies in this review found no significant differences in food intake with rare sugar administration. However, some studies reported that PSI consumption results in decreased food intake^(43,51,60,66)^, and there are several mechanisms of action by which rare sugars could potentially affect appetite.

Leptin plays an important role in long-term appetite regulation; it suppresses appetite and increases energy expenditure^(39)^. Six animal studies found significantly decreased leptin levels with PSI supplementation^(41,42,60,65,66,71)^. This could be explained by the decrease in body fat observed in each study, as leptin is mainly secreted by adipose tissue. The effect of rare sugars on leptin signalling in humans is not clear, with leptin levels found to increase^(39)^ or remain the same^(36)^ with daily PSI consumption despite significant reductions in body fat. Although generally correlated with adiposity, leptin levels show substantial inter-individual variation and are affected by inputs from the sympathetic nervous system, insulin levels and long-term dietary intake^(87)^. Further research is needed to investigate the potential effect of rare sugar intake on leptin signalling.

Appetite is also regulated by gut hormones such as GLP-1 and gastric inhibitory peptide which, in addition to their insulinotropic effects, slow gastric emptying. PSI has been shown to stimulate the release of GLP-1 in animal studies^(51,88)^, an effect which could induce satiety and reduce food intake. TAG has been found to stimulate GLP-1 release^(52)^ and slow gastric emptying^(52,89)^ in human trials, but the effect of rare sugar intake as part of a mixed meal has not to our knowledge been investigated.

Dietary monosaccharides are known to affect appetite-regulating peptides in the hypothalamus; elevated glucose or fructose consumption has been shown to reduce the expression of the appetite-suppressing signals peptide YY and pro-opiomelanocortin^(90)^. To our knowledge, the effects of rare sugars on hypothalamic appetite peptides have not been studied. Further work is needed to investigate the effects of different rare sugars on appetite, incretin release, gastric emptying and fullness in real-life conditions.

#### Effects on inflammatory markers and oxidative stress

Obesity, T2D and CVD all involve inflammation and increased oxidative stress^(83)^. A reduction in inflammatory cytokines and oxidative stress could therefore be a key mechanism by which rare sugars may slow the progression of these conditions. Several animal studies have found reduced markers of inflammation or oxidative stress when dietary sucrose or fructose was replaced with PSI^(71,73,82)^. Although in all of these studies the control group consumed more sucrose than the experimental group, the energetic intake was matched between groups so the reduction in inflammatory cytokines is unlikely to be a result of energetic restriction. In one study in which TAG (30 % solution), fructose (30 % solution) or plain drinking water were provided to mice over 24 weeks, significantly increased TNF*α* and IL-1*β* levels were reported with fructose intake^(69)^. There were also significant increases in these cytokines with TAG intake, but the increase was around half of that with fructose. As fructose provides around 16.7 kJ/g and TAG is estimated to provide 8.4 kJ/g^(5)^, these differences in inflammatory cytokine levels could be explained by differences in energetic intake between groups^(69)^. Studies using OLETF rats, a model for T2D, have found that PSI-treated animals had reduced fibrosis and fatty degeneration of pancreatic islets compared with control OLETF rats. This protective effect was attributed to the reduced release of pro-inflammatory cytokines in PSI-fed animals^(59,60)^. Similarly, the replacement of dietary sucrose with TAG has been found to reduce atherosclerosis in animals^(70,91)^, although once again the possible contribution of energetic restriction must be taken into account^(92)^.

#### Effects on the gut microbiome

The composition of the gut microbiota can be affected by dietary change, and it is becoming increasingly clear that changes in the gut microbiome are linked to a wide range of health-related factors such as inflammatory state and adiposity^(82)^. Probiotics and polyphenol-rich fruit extracts, which improve the diversity of the gut microbiome, have been shown to also reduce visceral adiposity and obesity^(45)^. The impact of rare sugars on the gut microbiome has only recently been studied, but the results from animal studies indicate that PSI intake can increase the proportion of species such as *Lactobacillus*, thought to be protective against fructose-induced NAFLD^(45)^. TAG, as it is poorly absorbed, can act as a prebiotic and has been shown to work synergistically with probiotics in reducing the susceptibility of mice to chemically induced colitis^(47)^. This potential for changes to the gut microbiome requires further exploration in human studies but should also be considered as a potential mechanism when interpreting the results of existing studies.

### Potential use of rare sugars as functional foods

Functional foods are those containing ingredients which exert positive health effects, and therefore have health-promoting properties besides their nutritional value^(17)^. The majority of the evidence suggesting health benefits from long-term rare sugar consumption comes from animal studies that, if replicated in humans, could provide significant health benefits. However, because of the very high-energy diets and large rare sugar dosages used in many of these trials, there is doubt about whether similar effects would be seen in humans. For example, the dramatic reduction in lipid accumulation in the liver seen in studies of PSI^(41–46,49)^ or ALL^(27)^ consumption suggests an application for rare sugars in preventing NAFLD. Only one of these studies^(46)^ provides daily food intake data, stating that the *ob/ob* mice in the experimental group consumed 3–4 g PSI per kg body weight per day, with control groups consuming an isoenergetic diet of normal CE2 pellet food. This quantity would equate to an intake of at least 210 g PSI per day for an average 70 kg human – a quantity clearly unrealistic for PSI consumption in foods.

In this review, the effects of long-term consumption in humans have been reported in only three studies using TAG in the USA and India^(37,38,40)^, two studies using PSI in Korea and Japan^(28,36)^ and one study in Japan using RSS^(39)^. Although these trials did report significant benefits from rare sugar consumption, it is important to note that they involved relatively large doses of rare sugars taken as daily dietary supplements. If rare sugars are to be promoted for their health benefits, research studies must take into account that they are more likely to be consumed in smaller quantities as part of reformulated food products.

The rare sugar TAG is currently used as a sweetener (branded ‘Tagatesse’) in products sold by Damhert Nutrition in Belgium, the Netherlands and Luxembourg^(10)^. A typical product, gluten-free spiced biscuits, contains 0·6 g TAG per 10 g portion. In contrast, participants in the 2015 trial conducted by Ensor *et al.* ^(40)^ consumed 15 g of TAG three times per day. Products containing PSI are also available, primarily in the USA, where items such as soft drinks, protein bars and cookies are sweetened with PSI^(11)^. For example, Quest hero bars, marketed as low-carbohydrate snacks, contain 11 g PSI per 60 g bar (along with erythritol and soluble fibre)^(93)^. This quantity is more comparable to the amounts used in clinical trials, for example, Han *et al.* ^(36)^ reported significant reductions in body weight and BFP when overweight participants consumed 7 g PSI twice daily for 12 weeks, compared with a control group consuming a sucralose placebo.

The cost of rare sugar production has been reduced by advances in biotechnology. The cost of PSI is now estimated at $7/kg, comparable with erythritol^(94)^. Recent advances in genetic engineering have produced yeasts that can generate TAG from whey waste from yogurt making, greatly reducing its cost^(7)^. Rare sugars are therefore becoming attractive alternatives to other sweeteners in the reformulation of products.

Another important consideration if rare sugars are to be used in the reformulation of foods is their sensory properties. Rare sugars tend to be slightly less sweet than sucrose but have similar sweetness profiles, suggesting that the temporal sweetness profile and sweetness quality may be similar to sucrose but the intensity will be lower^(14,95)^. When used in combination with sucrose, some rare sugars can provide desirable sensory characteristics while also reducing energy content^(14,95)^.

In considering the use of rare sugars within functional foods and in the reformulation of foods, it is also vital to consider the safety of long-term rare sugar intake. Both PSI and TAG have been given generally recognised as safe status^(8,9)^. In tolerance testing in healthy volunteers, the maximum single dose of PSI that resulted in no severe gastrointestinal symptoms was 0·4 g per kg body weight^(75)^, although Hayashi *et al.* ^(28)^ reported no evidence of toxicity with a single dose of PSI at 0·5–0·6 g per kg body weight. A large clinical trial investigating the safety and efficacy of TAG for treating patients with T2D reported no toxic effects on renal or hepatic function, although there were transient mild gastrointestinal symptoms^(37)^. One consideration in the assessment of the safety of sugars is their natural presence in a typical human diet. PSI exists in small amounts in wheat and Itea plants as a free sugar, but more substantial amounts (up to 135 mg/100 g) are formed when fructose undergoes cooking processes, such as in Worcester sauce, brown sugar, maple syrup, ketchup and cola^(15)^. TAG occurs naturally in Sterculia setigera gum, and small quantities have been found in sterilised and powdered cows’ milk, a variety of cheeses and other dairy products^(96)^. Nonetheless, it is vital to consider the effects of large-scale increases in the intake of these sugars in a population. The rare disaccharide trehalose appears naturally in small amounts in mushrooms, honey and other foods and was considered generally recognised as safe. However, when it begun to be widely used in the manufacture of baked goods and cereals, average intakes increased from less than 0·3 g/d to over 30 g/d. This change in nutrient availability led to the evolution of strains of the pathogenic bacterium *Clostridium difficile* which were able to utilise trehalose as an energy source, and therefore outcompete other gut microbiota^(97)^. The effects of increased intake of rare monosaccharides on the gut microbiome should be carefully considered before encouraging increased general intake of rare-sugar-containing products.

The generally recognised as safe status, suitable sensory characteristics and reasonable costs of both PSI and TAG make them attractive options as novel sugar replacers in the reformulation of food products. However, there is a need for further long-term human trials in different populations, using realistic dosages within real food matrices, with careful monitoring of adverse effects and impact on gut microbiome before any of the promising results from animal studies can be translated into health claims for rare sugars as functional foods.

### Implications for research

This review has highlighted gaps in the research on the use of rare sugars. Studies in this field have tended to focus on either postprandial glucose metabolism, long-term glycaemic control or lipid metabolism. As a result, there is a lack of research linking these different areas. Research to date has been predominantly conducted in animals, often using DIO animals consuming high-energy diets, for example, several studies^(41–45)^ involved PSI intake as 5 % of a weight-promoting diet (replacing sucrose), with control diets typically containing 20 % fat and 37 % sucrose by weight^(41)^. This is a widely used method to model obesity in rodents^(98)^ but may not accurately represent the complexities of energy metabolism in humans consuming an unhealthy diet. The small number of human studies to date has been carried out in limited populations (PSI and RSS primarily in East Asian subjects, TAG mainly in subjects with T2D). The details of how rare sugars are absorbed, metabolised and excreted in humans are not yet known. PSI and ALL are both found in human urine at levels higher than would be expected considering extremely low levels in the diet, highlighting gaps in our knowledge of their metabolism^(99)^. The mechanisms by which rare sugars exert their effects are not fully understood; therefore, it is not possible to draw conclusions on their potential health benefits in different populations. The majority of the human trials have involved rare sugar solutions as a supplement, and there is no evidence to date for health benefits of rare sugar consumption in reformulated products as part of a normal diet. In order for rare sugars to be classed as functional foods, robust evidence would be required demonstrating measurable improvements in health markers or outcomes from regular consumption of reformulated foods containing rare sugars.

One of the key differences in research methodology highlighted by this review is the nature of the control conditions. While many researchers took steps to reduce or eliminate the effect of differences in energetic intake between groups, some studies used rare sugars to replace other carbohydrates in the diet, and thus there was a difference in energy intake between groups. In some cases, this difference was substantial, for example, in one study in mice where all of the sucrose in a Western diet (34 % of the diet by weight) was replaced by TAG^(91)^. It is possible therefore that the reduced serum lipids, reduced atherosclerotic lesions and reduced adipose tissue weight observed in this study were partly a result of energetic restriction. Reduced energy intake can rapidly lead to decreased triglyceride levels in tissues including the liver, reduced visceral fat and increased insulin sensitivity in people with obesity^(92)^; therefore, it is vital that studies exploring similar effects with rare sugar intake ensure that experimental and control diets are isoenergetic. Overall food intake should also be monitored and reported. Several animal studies in this review reported reduced food intake with PSI^(43,46,51,60,62)^ or RSS^(66,74)^ supplementation, suggesting a possible effect on appetite. In long-term studies in people, where there may be large differences in energy intake between different participants, small changes in appetite with rare sugar intake could result in differences in energy intake which may not be detected even with careful dietary monitoring. Even in studies with isoenergetic diets and monitored food intakes, the type and quantity of carbohydrates in experimental and control diets should be considered carefully. Consumption of fructose and high fructose corn syrup is known to have detrimental effects on lipid metabolism and insulin sensitivity^(100)^ and has been linked to increased cardiovascular risk and diabetes prevalence^(101)^. It is important when examining the benefits of a rare sugar to consider whether rare sugar consumption is ‘better than nothing’ or only ‘better than other free sugars’.

Short-term studies in both healthy volunteers and subjects with T2D have demonstrated a reduction in PEBG when a single dose of PSI or TAG is consumed alongside a carbohydrate load^(28,29,31–34,52,57)^. However, it is not clear whether this effect persists when rare sugars are consumed regularly^(36,39)^. Additionally, the importance of the timing of rare sugar consumption relative to the carbohydrate load and the effects of rare sugar consumption on appetite have not to our knowledge been investigated in humans. These are important considerations if health claims are to be made for products containing rare sugars as replacements for free sugars.

Many animal studies^(36,39,41–46,48,49,59,62,63,66,71–74)^, and two studies in humans^(36,39)^, have reported reductions in body fat with PSI or RSS intake. This hypolipidaemic effect appears to be mediated by changes in the expression or activity of enzymes involved in lipid metabolism (see Fig. 3). Importantly, the *in vivo* effects of PSI tend to oppose the effects of insulin. It is possible that reduced circulating insulin is the primary factor leading to reduced lipogenesis and increased oxidation of fatty acids. However, there were no significant changes in fasting insulin levels in the human studies^(36,39)^, or in the animal studies in which it was measured^(62,63,70)^. While both PSI and TAG have been shown to stimulate GLP-1 release^(51,52,88)^, it is only in mice that this has been linked to increased insulin release^(51)^. It is possible that rare sugars may potentiate insulin release in the short term, while improving insulin sensitivity and thus reducing basal insulin levels in the longer term. Further large-scale, long-term trials in different human populations would help to clarify the effect of rare sugars on insulin secretion and shed light on the mechanisms for the hypolipidaemic effects of rare sugars.

### Limitations of this review

The process of a scoping review, as distinct from a systematic review, has certain limitations. In order to rapidly map the existing literature, inclusion criteria were broad and study selection was not subject to the quality assurance typical in a systematic review. The quality of individual studies has not been formally assessed, and some evidence has been extracted from abstracts of papers, so detailed methods cannot be examined.

When attempting to collate and report data from a range of different studies, there is necessarily a degree of over-simplification. This review has largely reported on significant effects and their direction, but has not attempted to quantitatively compare effect sizes. Studies are not always directly comparable because of differences in animal models or subjects, rare sugar dosages and timescales.

When considering the results of human trials, it is significant that most of the studies carried out to date have been in East Asian populations. Differences in genetics and habitual diet could limit the extent to which these results can be generalised to other populations. Additionally, most studies have involved the acute administration of rare sugars in drinks or syrups. The effects of rare sugars as part of a typical human diet, and in different food matrices, are largely unknown.

### Summary

This scoping review has summarised the research into the observed health benefits of rare sugars. The majority of research has focussed on PSI, but other rare sugars have been shown to have beneficial effects.

Rare sugars have been shown to improve glycaemic control and reduce body fat in human clinical trials as well as in animal studies. The effect of lowering postprandial glucose levels could lead to multiple health benefits, and rare sugars may also affect other pathways linked to obesity, T2D, NAFLD and CVD, for example, by altering lipid metabolism, improving the gut microbiome or reducing inflammation. Therefore, the consumption of rare sugars, whether as sugar replacers, dietary supplements or in functional foods, could potentially provide health benefits. However, the number and scale of human studies is still limited, and the dosage, timing and frequency of consumption required to see beneficial effects in humans are not known. There are questions to be answered about the long-term efficacy of rare sugars and their effects on health outcomes in different populations. A clearer understanding of the absorption and metabolism of rare sugars in humans, their effects when consumed in realistic doses as part of reformulated foods and their mechanisms of action is vital when considering the potential benefits of rare sugars in the human diet.

## Supplementary material

For supplementary material accompanying this paper visit https://doi.org/10.1017/S0007114521003524. click here to view supplementary material
