# Supplementary material for: Rare sugars: metabolic impacts and mechanisms of action: a scoping review
Source: Br J Nutr. 2021 Sep 10;128(3):389–406. doi: 10.1017/S0007114521003524 (PMC9343225; doi:10.1017/S0007114521003524)
Supplement: Supplementary file 1 [file S0007114521003524sup001.docx]

**Table S1: Search Terms and Results of Literature Searching**

| **Search** | **Search terms** | **Scopus** | **Web of Science** | **PubMed** |
| --- | --- | --- | --- | --- |
| 1 | D-psicose OR psicose OR D-psi OR allulose OR D-allulose | 551 | 687 | 303 |
| 2 | D-tagatose OR tagatose OR D-tag | 650 | 688 | 335 |
| 3 | D-sorbose or sorbose OR D-sor | 529 | 499 | 282 |
| 4 | D-allose OR allose | 381 | 437 | 246 |
| 5 | ‘rare sugar’ OR ‘novel sweetener’ OR ‘new sweetener’ OR ketohexose OR aldohexose | 77 | 536 | 404 |
| 6 | ?Glyc?emi* OR ‘blood sugar’ OR insulin OR diabetes OR HbA1c | 356,952 | 680,350 | 703,422 |
| 7 | Lipid OR adipose OR fat OR ‘body composition’ OR obesity OR lipo* OR NAFLD OR NASH OR ‘fatty liver’ | 80,712 | 1,301,826 | 1,264,128 |
| 8 | ‘Cardiovascular disease’ OR CVD OR ‘heart disease’ OR stroke OR atherosclerosis | 503,577 | 700,494 | 537,047 |
| 9 | Antioxidant OR ‘oxidative stress’ OR redox OR ‘reactive oxygen species’ OR ‘free radical’ OR thioredoxin OR glutathione | 42,011 | 846,983 | 706,203 |
| 10 | 1 AND (6 OR 7 OR 8 OR 9) | 69 | 333 | 87 |
| 11 | 2 AND (6 OR 7 OR 8 OR 9) | 40 | 272 | 79 |
| 12 | 3 AND (6 OR 7 OR 8 OR 9) | 12 | 110 | 70 |
| 13 | 4 AND (6 OR 7 OR 8 OR 9) | 23 | 213 | 84 |
| 14 | 5 AND (6 OR 7 OR 8 OR 9) | 20 | 147 | 117 |
| **Unique references identified in database** | | **123** | **613** | **425** |

**Table S2a: Summary of included *in vivo* animal studies.**

| **Ref** | **Authors** | **Location** | **Animal model** | **Sugar used** | **Timescale and dosage** | **Overview of methods** | **Outcome measures** | **Key results and conclusions** |
| --- | --- | --- | --- | --- | --- | --- | --- | --- |
| 25 | Oku et al., 2014 | Japan | Wistar rats | SOR | 0.0495g single oral dose. | Test solutions (0.45g sucrose, 0.45g sucrose + 0.0495g SOR, 0.45g sucrose + 0.0495g L-SOR) given orally in solution, plasma glucose and insulin measured every 30 minutes for 3 hours. | Plasma glucose and insulin. | With SOR: elevation of plasma glucose was suppressed after 30 and 60 minutes. Plasma insulin was also lower at 30 and 60 minutes in SOR group compared to sucrose-only group. |
| 26 | Yamada et al., 2014 | Japan | Sprague-Dawley rats | SOR | 3% of diet for 28 days. | 2 groups (n=7) given control diet or 3% SOR (replacing cornstarch) diet ad libitum for 28d. BW monitored. At sacrifice, non-fasting blood collected for biochemical analysis and organ weights measured. | BW and composition, organ and tissue weights, serum insulin, glucose, lipids and biochemical parameters. | No significant differences observed in BW, food intake or adipose tissue weights. Cecum weight increased and cecal pH reduced in SOR group.  Serum insulin reduced, but no significant change in serum glucose, in SOR group. Uric acid and aspartate aminotransferase reduced in SOR group. No other significant differences observed. |
| 27 | Yamamoto et al., 2017 | Japan | STAM mice (C57BL/6J injected with STZ then fed high fat diet to induce NASH) | ALL | 2% of high fat diet for 3 weeks. | 2 groups (n=5-8) of STAM mice given high fat diet or high fat diet with 2% ALL. Control group of C57BL/6J mice given normal diet. BW monitored. At sacrifice, organ weights and serum biochemical parameters measured. Liver sections examined microscopically. | BW, liver weight. Serum aspartate aminotransferase (AST), alanine aminotransferase (ALT), triglyceride and glucose. Liver injury (NAFLD activity score) | STAM mice had reduced BW, increased liver weight, and 7-fold higher fasting serum glucose compared to control mice. No significant differences were observed between ALL-fed STAM and control STAM groups. Serum ALT and AST were increased in STAM mice compared to control, indicating liver injury. ALT level was reduced in ALL-fed STAM group compared to control STAM group, but was still significantly higher than control group. No significant differences in serum triglyceride were seen between any groups.  Hepatic lipid accumulation was increased in STAM mice compared to control, but in STAM mice given ALL this was completely suppressed. Scores of hepatic pathology were reduced in ALL-fed STAM mice compared to the STAM group. |
| 41 | Do et al., 2019 | Korea | Male C57BL/6J mice | PSI | 5% of high fat diet for 8 weeks. | 2 groups (n=8), given either a high fat diet with 5% PSI (replacing sucrose), or an isoenergetic amount of high fat diet. BW monitored. At sacrifice body composition and organ weights measured, plasma, hepatic and fecal lipids, plasma adipokines and cytokines and hepatic enzyme activities measured. Liver histopathology examined. | BW and composition, feed efficiency ratio, plasma adipokines and cytokines, plasma, hepatic and fecal lipids, organ weights and histopathology, hepatic enzyme activities. | With PSI: lower weight gain and feed efficiency ratio. Reduced adipose tissue. Reduced liver weight, increased kidney and muscle weight. Reduced plasma glucose, resistin and leptin. Reduced fecal fatty acids and triglycerides, reduced plasma triglycerides, reduced hepatic triglycerides, fatty acids and cholesterol. Reduced activities of G6PDH, PAP, ACAT but no significant difference in FAS or enzymes involved in β-oxidation. Smaller and fewer lipid droplets in liver, smaller adipocytes. |
| 42 | Han et al., 2016 | Korea | Male C57BL/6J mice | PSI | 5% of high fat diet for 16 weeks. | 6 groups (n=10), given normal diet (control), high fat diet (control) or high fat diet with 5% PSI, erythritol, glucose or fructose (replacing sucrose). BW monitored. At sacrifice body composition measured, plasma, hepatic and fecal lipids, plasma adipokines and hepatic enzyme activities measured. | BW and composition, plasma adipokines, plasma, hepatic and fecal lipids, hepatic enzyme activities. | With PSI: lower weight gain and feed efficiency ratio. Reduced plasma leptin and resistin. Reduced liver weight, increased kidney and muscle weight. Reduced white adipose tissue. Values in PSI group close to normal diet control. Reduced plasma triglycerides, total cholesterol and LDL-cholesterol. Reduced hepatic free fatty acids, triglycerides and cholesterol. Reduced fatty acid synthase activity and increased β-oxidation activity in adipose tissue. |
| 43 | Hossain et al., 2011 | Japan | OLETF rats | PSI | 5% solution in drinking water for 14 weeks. | OLETF rats (n=15 per group) given plain water, 5% PSI solution or 5% glucose solution. Blood glucose measured periodically, OGTT at 14 weeks, liver and pancreas histology at sacrifice, immunostaining of liver tissue for glucokinase (GK). | BW and composition, AUC for glucose and insulin in OGTT, hepatic steatosis, pancreas morphology. | With PSI: lower weight gain and food intake. Reduced abdominal fat. Reduced AUC for glucose and insulin. Although periodical blood glucose increased gradually in OLETF control and glucose-fed groups, it was static in LETO control and PSI-fed OLETF rats from 3 weeks.  GK translocation to cytoplasm impaired in OLETF control rats, but no significant difference between LETO control, PSI-fed and glucose-fed groups. |
| 44 | Han et al., 2020 | Korea | Male C57BL/6J mice | PSI | 5% of high fat diet for 16 weeks. | 4 groups (n=10) given normal diet, high fat diet, high fat diet + 5% PSI or 5% erythritol (substituted for sucrose). BW monitored and energy expenditure measured. At sacrifice body composition measured, plasma lipids and adipokines measured, gut microbiota and short chain fatty acids measured. | BW and composition, plasma lipid profile, plasma adipokines, energy expenditure, gut microbiota and short chain fatty acids.  Liver histopathology. | With PSI: lower BW compared to high fat diet and erythritol groups. Adipose tissue weight dramatically reduced compared to high fat diet group. Total cholesterol, HDL and non-HDL cholesterol decreased compared to high fat diet. Fatty acid synthase activity reduced and β-oxidation activity increased in white adipose tissue. Significant increase in energy expenditure. PSI and normal diet groups showed higher diversity in gut microbiota than high fat diet group.  Reduced accumulation of lipid droplets in hepatic tissue with PSI compared to high fat diet group. Fibrotic tissue absent in normal diet and PSI groups, present in high fat diet and erythritol groups. |
| 45 | Han et al., 2020 | Korea | Male C57BL/6J mice | PSI | 5% of high fat diet for 16 weeks. | 3 groups (n=9) given normal diet, high fat diet or high fat diet + 5% PSI (substituted for sucrose). BW monitored. At sacrifice body composition and plasma inflammatory markers measured. Expression of genes in liver and white adipose tissue measured. | BW and composition, plasma inflammatory markers, up/downregulation of gene expression in liver and white adipose tissue, gut microbiota. | With PSI: reduced BW and adipose tissue weight, no significant difference in food intake. Reduced hepatic lipid content. PSI supplementation reversed the differences in organ weights seen with high fat diet. Groups of up/downregulated genes identified as associated with obesity-related inflammation, reduced to near normal-diet levels with PSI. PSI diet increased beneficial bacteria Lactobacillus and Coprococcus. |
| 46 | Itoh et al., 2015 | Japan | C57BL/6J mice (*ob/ob*  and wild-type) | PSI | 2.5% or 5% of diet for 15 weeks. | 3 groups (n=14) of *ob/ob* mice given 0, 2.5 or 5% PSI, control group (wild-type) given normal diet. BW monitored, hepatic steatosis and fat deposition measured by MRI. Body composition and histological examination at sacrifice. | BW and composition, hepatic steatosis. | BW gain lower in 5% PSI group than in *ob/ob* control. Liver weight, visceral fat and fat mass lower in PSI groups than *ob/ob* control, with no difference in fat-free mass. Livers of *ob/ob* mice showed signs of hepatic steatosis not present in wild-type mice- this was inhibited in 5% PSI group. |
| 47 | Son et al., 2019 | Korea | BALB/c mice | TAG | 25mg every other day for 3 weeks, with or without 109 cfu/ml *Lactobacillus rhamnosus GG* (LGG), by oral gavage. | 4 treatment groups (n≥3) given PBS, TAG, LGG or LGG+TAG for 3 weeks. In 3^rd^ week dextran sulphate sodium (DSS) added to drinking water to induce colitis. | BW, food intake and colitis disease activity index assessed daily, fecal microbiota analysed and classified.  At sacrifice, morphological and histological analysis of colon undertaken, serum IL-6, IL-10 and TNFα determined. | DSS induced colitis symptoms (reduced BW, diarrhoea, bloody stools), as well as reduced colon length, irregular crypt structure, inflammatory cell infiltration, increased serum IL-6, IL-10 and TNFα. Group given LGG+TAG were less susceptible to colitis than LGG group or TAG group- had higher BW, higher food intake and reduced diarrhoea scores. BW was higher in TAG, LGG and LGG+TAG groups than in DSS group. In all 3 ‘treatment’ groups, the reduced colon length and acute inflammation seen with DSS treatment were attenuated, with a synergistic effect with LGG+TAG. All 3 treatments reduced serum IL-6 and IL-10, but only LGG+TAG reduced serum TNFα. The effects of DSS-induced colitis on the intestinal bacterial communities were reduced in the LGG+TAG group. |
| 48 | Chen et al., 2017  ABSTRACT ONLY | China | Wistar rats | PSI | 5% of diet for 4 weeks. | 5 groups given 5% glucose, fructose, cellulose or PSI. At sacrifice, blood lipid profile, tissue morphology and genes involved in lipid metabolism measured. | BW, body fat, plasma lipid profile, expression of genes related to lipid metabolism. | With PSI: lower weight gain, reduced epididymal fat, smaller adipocyte size, improved blood lipid profile and antioxidant level. Increased expression of succinate dehydrogenase and hepatic lipase. |
| 49 | Chen et al., 2019^)^ | China | Male Wistar rats | PSI | 5% of diet for 4 weeks. | 5 groups (n=6) given AIN-76A diet or the same diet with CHO partially replaced with 5% glucose, fructose, cellulose or PSI. BW measured every two days. At sacrifice body fat, plasma lipids and hepatic gene expression measured. | BW, body fat, plasma lipid profile, hepatic gene expression. | With PSI: lower weight gain, reduced abdominal and epididymal fat, reduced plasma triglyceride, free fatty acids and LDL cholesterol compared to control. Increased hepatic expression of catalase and succinate dehydrogenase. |
| 50 | Pratchayasakul et al., 2020  ABSTRACT ONLY | Thailand | Rats | PSI | 1.9g per kg BW per day for 12 weeks | Rats (n=56) fed control diet or HFD for 12 weeks. HFD-fed rats then given PSI or metformin for 12 weeks. Cognition and brain parameters determined at 24 weeks. | Brain oxidative stress, mitochondrial dysfunction, microglial hyper-activation, apoptosis, insulin insensitivity, hippocampal synaptic dysfunction, cognitive decline. | All stated cognition and brain parameters were observed in HFD rats. Both PSI and metformin attenuated brain oxidative stress, mitochondrial reactive oxygen species production and hippocampal apoptosis and improved learning. Metformin gave greater improvement than PSI in brain mitochondrial dysfunction and microglial hyper-activation, and improved both learning and memory. |
| 51 | Iwasaki et al., 2018 | Japan | C57BL/6J mice  (HFD-induced obese, *GLP1r* knockout) | PSI | 0.3, 1 or 3g per kg BW single dose.  1g per kg BW per day for 10 days. | PSI given orally or by intraperitoneal injection. Food intake monitored. Blood glucose, insulin and GLP-1 measured before and after intraperitoneal glucose injection.  PSI given orally at onset of light or dark period. Food intake monitored, blood glucose and insulin measured before and after intraperitoneal glucose injection. | Food intake, GLP-1 secretion, response in glucose tolerance test. | Oral PSI administration decreased food intake for 6 hours without aversion. Cumulative intake normalised after 24 hours. This effect was not seen in *GLP1r-*knockout mice, nor when PSI given by injection. Oral PSI administration 60 minutes before glucose injection attenuated increases in blood glucose at 15, 30 and 60 minutes without affecting basal blood glucose, and increased insulin release at 15 minutes.  Daily oral PSI administration at onset of light period reduced food intake during light period, reduced fasting blood glucose and attenuated hyperinsulinaemia in diet-induced obese mice. Blood glucose and insulin after glucose injection were also reduced. Effects were not significant in *GLP-1r* knockout mice, nor if PSI was given at onset of dark period. |
| 54 | Yagi and Matsuo, 2009 | Japan | Wistar rats | PSI | 3% of diet for 12-18 months. | 2 groups (n=18) given diet with 3% sucrose or D-psi ad libitum. BW, food intake and symptoms of toxicity monitored. At sacrifice, organ weight and morphology examined, blood biochemical parameters measured. | BW, organ weight and morphology, haematological and biochemical measurements. | BW not significantly different between groups at 12 months but reduced in PSI group at 18 months. With PSI: liver and kidney weights increased at 12 and 18 months, brain, lung and pancreas and cecum weights increased at 18 months, intra-abdominal adipose tissue weight reduced at 18 months. No significant differences in chemical values. Fatty degeneration & hepatocellular fibrosis observed in PSI but not sucrose group, slight increase in pathological lesions in liver at 18 months. Overall effects not suggestive of overt PSI toxicity. |
| 58 | Baek et al., 2010 | Korea | Male C57BL/6J *db/db* mice | PSI | 200mg per kg BW for 4 weeks. | 4 groups of *db/db* mice (n=10) given water, PSI, D-glucose or fructose orally. Wild-type control group given water. BW, plasma glucose and insulin, plasma, liver and fecal lipids measured. OGTT at 28 days. | BW, plasma glucose and insulin, AUC for glucose in OGTT, lipid profiles. | With PSI: lower weight gain, lower plasma glucose compared to all groups. No significant difference in AUC for glucose in OGTT compared to control. Lower liver triglyceride and total cholesterol, no significant difference in plasma or fecal lipids. |
| 59 | Hossain et al., 2012 | Japan | OLETF rats | PSI | 5% solution in drinking water for 13 weeks. | OLETF rats (n=15 per group) given plain water, 5% PSI solution or 5% glucose solution. BW and composition monitored. Periodical fasting blood glucose and OGTT measurements. At sacrifice serum adipokines measured and adipose tissue and pancreas morphology examined. | BW and composition, AUC for glucose in OGTT, degree of insulin resistance (HOMA), histopathology of pancreatic islets, adipose tissue morphology. | With PSI: lower weight gain with no change in food intake. Reduced total fat and % fat mass. Reduced fasting blood glucose and AUC for glucose in OGTT. Reduced insulin resistance. OLETF control group had evidence of fibrosis and fatty degeneration in islets, absent in PSI-fed group. |
| 60 | Hossain et al., 2015 | Japan | OLETF rats | PSI | 5% solution in drinking water for 60 weeks. | OLETF rats (n=10 per group) given 5% PSI solution or normal drinking water. LETO control rats fed normal diet. Fasting and postprandial blood glucose measured periodically. OGTT, plasma lipids, adipokines and cytokines measured. At sacrifice adipose tissue and pancreas morphology examined. | AUC for glucose in OGTT, degree of insulin resistance (HOMA), plasma lipids, cytokines and adipokines, histopathology of pancreatic islets, adipose tissue morphology. | With PSI: reduced postprandial blood glucose from 35 weeks. Reduced plasma insulin and reduced insulin resistance (PSI-fed OLETF similar to LETO rats). Reduced inflammatory cytokines. Fibrotic, disorganised islets observed in OLETF control group, much less prominent and severe in PSI-fed group. |
| 61 | Kanasaki et al., 2019 | Japan | Golden Syrian hamsters | PSI | 3% of normal diet or high fat diet for 8 weeks. | 2 groups (n=8) given normal diet with or without 3% PSI. 2 groups (n=8) given high fat diet for 4 weeks then high fat diet with or without 3% PSI for 4 weeks. BW monitored, serum glucose, insulin, lipids and PCSK9 measured. | BW, serum glucose, insulin, lipids and PCSK9. | Normal diet hamsters: no significant differences in BW, serum glucose or insulin. Reduced cholesterol in VLDL and medium LDL, increased cholesterol in very small LDL and HDL, reduced LDL/HDL ratio.  High fat diet hamsters: Reduced cholesterol in LDL, particularly medium and small LDL. Reduced LDL/HDL ratio.  Both groups had reduced serum PCSK9 with PSI compared to control. PSI seems to improve cholesterol metabolism, possibly by reducing serum PCSK9. |
| 62 | Ochiai et al., 2013 | Japan | Wistar rats | PSI | 5% of diet for 8 weeks. | Rats fed high sucrose diet for 7 weeks, then 4 groups (n=8) given high starch diet with PSI (NP) or cellulose (NC) or high sucrose diet with PSI (SP) or cellulose (SC). BW monitored, serum lipids, insulin and leptin measured, liver glycogen and lipids measured at sacrifice. | BW, serum lipids, insulin and leptin, hepatic CHO and lipid content. | With PSI, reduced BW gain and adipose tissue weight. For rats on high sucrose diet, PSI reduced serum HDL and LDL cholesterol and increased non-esterified fatty acids. No significant differences in serum glucose, insulin or leptin. Hepatic triglyceride and cholesterol higher with PSI in high-starch diet groups, but no significant differences in hepatic glycogen. |
| 63 | Ochiai et al., 2017 | Japan | Wistar rats | RSS or modified glucose syrup (MGS) | 30% of diet for 8 weeks. | 4 groups (n=8) given high sucrose control, HFCS, RSS or MGS in ad libitum diet. Sucrose (approx. 30% of diet w/w) replaced with HFCS, RSS (PSI 1.5%, SOR 2.9%, TAG/ALL 1.1% of diet) or MGS (PSI 3.3% of diet) for 8 weeks. Food intake and BW monitored. At sacrifice, organ weights recorded and biochemical analysis of serum conducted. | BW & composition, serum fasting glucose, insulin, triglycerides, total cholesterol. Liver lipid profile. | With RSS, reduced adipose tissue weight and body fat percentage compared with sucrose group. Reduced food efficiency with RSS and MGS. Increased liver and kidney weights with both RSS and MGS compared to sucrose.  No significant differences in blood biochemical parameters. |
| 64 | Pongkan et al., 2020 | Thailand | Male Wistar rats | PSI | 1.9g per kg BW per day (3% solution in drinking water) for 12 weeks | 4 groups (n=6). 3 groups fed HFD for 12 weeks (control group fed ND). HFD groups then given PSI, metformin (300mg/kgBW/day) or sterile drinking water for 12 weeks. Cardiac function measured at 12 and 24 weeks. Plasma glucose, insulin and lipids measured and insulin resistance estimated, OGTT carried out, markers of cardiac dysfunction measured at 24 weeks. | Electrocardiograph for cardiac function, heart rate variability, cardiac mitochondrial function and oxidative stress, plasma glucose, insulin and cholesterol, homeostasis model assessment of insulin resistance, plasma and cardiac malondialdehyde. | No significant difference in food intake per body weight, but HFD animals had increased body weight and visceral fat. No significant difference in body weight or visceral fat with PSI compared to HFD group. PSI and metformin both attenuated insulin resistance, metformin improved lipid profile but no significant difference in lipids with PSI. Both PSI and metformin improved cardiac function, reduced cardiac oxidative stress, reduced evidence of cardiac mitochondrial dysfunction and reduced levels of cardiac apoptotic proteins compared to HFD group. |
| 65 | Nagata et al., 2015 | Japan | Sprague-Dawley rats | PSI | 3% of diet for 4 weeks. | 2 groups (n=24) given control diet or 3% PSI diet for 4weeks then 5-6 animals sacrificed every 6h over 24h without fasting. 2 groups (n=8) fed as above and energy expenditure measured over 24hrs. | Serum glucose, insulin, lipids and leptin, hepatic enzyme activity, gene expression in liver, small intestine, muscle and adipose tissue. Total energy expenditure, fat oxidation and CHO oxidation. | With PSI: reduced BW and food intake. Reduced serum insulin, leptin and total cholesterol at some timepoints, no significant difference in serum glucose.  With PSI: decrease of G6PDH and ME (sig at 9am). Expr of PPARa sig higher in liver, Exp of MTP sig lower. . Total EE per kg BW sig higher during late light period Fat ox sig enhanced and carb ox sig reduced during dark period. |
| 66 | Iida et al., 2013 | Japan | Male Wistar rats | RSS | 30% of diet (replacing 50% of starch) for 8 weeks. | 3 groups (n=10), given diets containing 60% starch, 30% starch:30% HFCS or 30% starch:30% RSS. BW monitored. At sacrifice, body composition measured, fasting blood taken and plasma glucose, insulin, lipids and adipokines measured. | BW and composition, plasma glucose, insulin, lipids and adipokines. | With RSS: Lower weight gain, reduced intra-abdominal fat (dose-dependent effect).  Fasting blood glucose reduced in HCFS and RSS groups compared to starch group. Plasma insulin reduced in RSS group compared to HCFS and starch. No differences in plasma total cholesterol or triglycerides. Leptin in RSS group lower than starch, in HCFS group higher than starch. |
| 67 | Iga et al., 2010 | Japan | Wistar rats | ALL | Acute administration study: 15-25g per kg BW in single dose.  Subchronic feeding study: 0-3% in food for 6 months. | Acute administration study: rats (n=4 per group) given single dose after 12h fast, fasted for 12h then observed for 48h.  Subchronic feeding study: rats (n=10 per group) given ALL in food. BW and food intake monitored. At sacrifice, haematological parameters and tissue weights measured. | Acute administration study: number of deaths, LD50.  Subchronic feeding study: BW, food intake, haematology, organ and tissue weights. | Acute administration study: LD50=20.5g per kg BW.  Subchronic feeding study: BW and feed efficiency ratio reduced in 3% ALL group compared to control. Lungs, soleus and gastrocnemius muscle weights reduced in 3% ALL group. No significant differences in haematological parameters. ALL concluded to be non-toxic in rats. |
| 68 | Nagata et al., 2018 | Japan | Sprague-Dawley rats | PSI, TAG, SOR | 3% of diet for 4 weeks. | 5 groups (n=6) given AIN-93G diet with or without 3% rare sugar or fructose replacing cornstarch. Sacrificed without fasting. Serum and fecal lipids, hepatic enzyme activity and gene expression in liver and small intestine measured. | Serum lipids, faecal lipids, hepatic enzyme activity, gene expression in liver and small intestine. | No significant differences in BW, feed efficiency ratio or liver weight. TAG-fed group had increased serum free fatty acids compared to fructose-fed group. Faecal excretion of fatty acids was decreased by PSI but increased by SOR.  Activity of fatty acid synthase was decreased by PSI but increased by TAG. G6PDH and PAP expression was decreased in PSI-fed group compared to control. Rare sugars affect lipid metabolism differently in rats. |
| 69 | Collotta et al. 2018 | Italy | C57BL/6J mice | TAG | 30% of solid diet or 30% syrup drink for 24 weeks. | 5 groups (n=6) given a 30% TAG or fructose solid diet, a control diet with 30% TAG or fructose syrup or a control diet with water. BW and glycaemia monitored. Plasma lipids, adipokines, cytokines and markers of myocardial oxidative stress measured. | BW, fasting glucose, HbA1c. Plasma lipids, leptin, inflammatory markers and MDA (marker of myocardial oxidative stress) measured. | Fructose-fed groups had higher weight gain, increased plasma fasting glucose and %HbA1c, increased leptin, serum triglyceride and LDL and decreased HDL, and a 3-fold increase in MDA. No significant differences were seen with TAG compared to control. Both fructose and TAG caused increased TNF-α and IL-1β, but the increase with TAG was half that of fructose. |
| 70 | Police et al., 2009 | USA | C57BL/6J  *LDLr-/-* mice | TAG | 30% of diet for 16 weeks. | 2 groups (n=12, equal m/f) given TAG or sucrose diet for 16weeks. Control (n=10) given standard murine diet. TAG/sucrose introduced gradually over 3 weeks before 16 week feeding period. BW and food intake measured. At sacrifice, organ weights measured, blood biochemistry analysed. Aorta and adipose tissue morphology examined. | BW and composition, fasting blood glucose, plasma lipids and lipoproteins. Atherosclerotic lesion area of aorta, morphology of adipose tissue, macrophage infiltration. | High sucrose diet group had increased BW, energy intake, adipose tissue mass and adipocyte size, TAG-fed group similar to control. Total cholesterol, VLDL and LDL increased with both sucrose and TAG compared to control, levels in the sucrose group higher than the TAG group.  Atherosclerotic lesion area increased with sucrose compared to TAG and control, and in females in the TAG group compared to control. Macrophage positive immunostaining seen in adipose tissue and aortic root of sucrose but not control or TAG groups. TAG as a CHO source leads to increased total cholesterol and atherosclerosis, but significantly less than sucrose. |
| 71 | Choi et al., 2018 | Korea | Male C57BL/6J mice | PSI | 3% of diet (PSI substituted for sucrose in high fat diet), with or without probiotics, for 12 weeks. | 7 groups (n=10), given high fat diet and two different probiotics with or without PSI. Control group given normal diet. At sacrifice, after 16h fast, BW and composition, plasma lipids, adipokines and cytokines, hepatic lipids, enzyme activity and gene expression measured. | BW and composition, plasma and hepatic lipids, plasma adipokines and cytokines, hepatic enzyme activities & expression. | With PSI: lower weight gain, reduced white adipose tissue. PSI and probiotics worked synergistically. Reduced plasma leptin, resistin and IL-1β in all groups fed PSI. Reduced activities of enzymes involved with fatty acid synthesis; increased activity of enzymes involved in β-oxidation in all groups fed PSI. |
| 72 | Chung et al., 2012 | Korea | Male Sprague-Dawley rats | PSI | 2.5 or 5% of diet for 52 days. | Rats (n=10 per group) fed high fat diet for 4weeks to induce obesity, then either switched to normal diet or kept on high fat diet with 5% sucrose, 5% eryrthritol or 2.5/5% PSI for 52 days. BW monitored, at sacrifice plasma lipids, body composition and organ weights measured. | BW and composition, feed efficiency ratio, plasma lipids, organ weights and histopathology. | With PSI: lower weight gain (dose-dependent), food efficiency ratio and fat accumulation (greater effect in animals fed normal diet). Increased serum total cholesterol, LDL-cholesterol and HDL-cholesterol and liver weight in PSI-ND group compared to ND group. No apparent differences in liver histopathology. |
| 73 | Kim et al., 2017 | Korea | C57BL/6J *ob/ob* mice | PSI | 5% of diet (replacing sucrose) for 12 weeks. | 2 groups (n=15) given AIN-93G diet or same diet with half of sucrose replaced with PSI (equivalent to 5% of diet). BW monitored, at sacrifice body composition, plasma lipids measured, adipose tissue morphology and gene expression examined. | BW and composition, plasma lipids, adipose tissue histology and gene expression. | With PSI: reduced final BW, white adipose tissue weight and adipocyte size. Lower plasma total cholesterol, LDL cholesterol and LDL/HDL ratio. No significant differences in triglycerides, free fatty acids or HDL cholesterol. Reduced expression of markers of inflammation (TNFα, IL-6, MCP-1) and adipogenesis (PPARs, SREBP-1c, LPL, FAS), increased expression of markers of lipolysis (HSL) and beta oxidation (CPT-1). |
| 74 | Shintani et al., 2017 | Japan | Wistar rats | RSS | RSS diluted to give 7% fructose (equates to around 1.4% PSI in solution) in drinking water for 10 weeks. | 3 groups (n=10), given water, HFCS or RSS. BW monitored. OGTT (2g per kg BW glucose) and insulin tolerance test carried out at 8 weeks. At 10 weeks, 4 rats per group sacrificed before and 30 min after a glucose load and livers examined for glucokinase translocation. At sacrifice, tissue weights measured and liver analysed for glycogen content. | BW and composition, AUC for glucose and insulin in OGTT, hepatic glucokinase distribution before and after glucose load, liver glycogen content. | With RSS: reduced weight gain, decreased total abdominal fat compared to control. (HFCS had no effect on BW but increased abdominal fat).  RSS decreased AUC for glucose and insulin in OGTT compared to control (AUC for glucose was increased by HFCS). Insulin sensitivity increased by RSS (decreased by HFCS).  Hepatic glycogen before glucose load was 3-fold higher with RSS than control or HFCS. Glycogen in HFCS group was reduced after glucose load compared to control and RSS. GK translocation to cytoplasm was increased with RSS compared to HFCS or control, both before and after glucose load. |
| 76 | Huang et al., 2018  ABSTRACT ONLY | China | Wistar rats | PSI | unknown | 5 groups (n=?), given normal diet or supplemented with PSI, cellulose, glucose or fructose. | BW, plasma lipids, liver histology, hepatic gene expression. | With PSI: reduced BW, serum triglycerides, free fatty acids and LDL-cholesterol. Increased expression of PPARα, reduced expression of fatty acid synthase. |
| 78 | Williams et al., 2013 | USA | Male Sprague-Dawley rats | TAG | 0, 0.6, 2 or 6g per kg BW, single dose by oral gavage along with ^14^C-labelled fructose | 5 groups (n=8) given oral gavage containing 2g/kg ^14^C-labelled fructose and varying doses of TAG. Blood samples from femoral vein catheter at regular intervals for 60 mins. | Blood glucose, plasma scintillation count to determine ^14^C fructose. | 2 and 6g/kg TAG reduced AUC of fructose absorption by 26 and 30% respectively.  No difference in blood glucose with 0.6 or 2g/kg TAG, but 6g/kg TAG caused increased blood glucose from 30 mins (this attributed to stress caused by malabsorption effects from large doses of fructose and TAG) |
| 88 | Hayakawa et al., 2018 | Japan | Male C57BL/6J mice | PSI | 0.5 – 2g per kg BW single oral dose. | PSI, resistant maltodextrin, dextrin, fructose or water administered orally, with or without inhibitors of glucose/fructose transport. Plasma GLP-1, portal GLP-1 and GIP measured. Luminal contents measured at 60 and 150min after administration. | Plasma and portal GLP-1 and GIP, rate of PSI absorption, with and without inhibitors of glucose/fructose transport. | Oral PSI administration increased plasma GLP-1 in dose-dependent manner, and stimulated GLP-1 but not GIP in the portal vein. Intraperitoneal injection of PSI did not stimulate GLP-1. PSI absorption was slower than glucose (25% PSI remaining in stomach after 60 mins compared to 2.6% with glucose). Inhibitors of SGLT1 and sweet receptor did not lower PSI-induced GLP-1 secretion. |
| 91 | Williams et al., 2015 | USA | ApoE knockout mice | TAG | 34% of diet by weight (entirely replacing sucrose) for 8 weeks. | 5 groups (n=8), given standard diet, Western diet (high fat & sucrose), Western diet with TAG, Western diet with BSN723 or Western diet with TAG and BSN723. BW monitored. At sacrifice body composition and evidence of atherosclerotic plaques measured. | BW and composition, serum lipids, extent of atherosclerosis. | TAG groups on Western diet showed lower BW gain than control Western diet group. Addition of TAG prevented the increase in adipose tissue caused by Western diet. Surface area of atherosclerotic lesions was greater in Western diet compared to standard diet; this was inhibited in TAG groups. The increase in serum cholesterol as a result of Western diet was significantly less in the groups receiving TAG. |

Reported differences are statistically significant unless stated otherwise. BW: body weight, PSI: D-psicose, TAG: D-tagatose, SOR: D-sorbose, ALL: D-allose, RSS: rare sugar syrup, HFCS: high fructose corn syrup, CHO: carbohydrate, AUC: area under curve, OGTT: oral glucose tolerance test, LDL: low density lipoprotein, HDL: high density lipoprotein, HFD: high fat diet, TNF-α: tumour necrosis factor α, NAFLD: non-alcoholic fatty liver disease, NASH: non-alcoholic steatohepatitis, ND: normal diet, IL: interleukin, OLETF: Otsuka Long-Evans Tokushima Fatty, LETO: Long-Evans Tokushima Otsuka.

**Table S2b: Summary of included studies in human subjects**

| **Ref** | | **Authors** | **Location** | **Study population** | **Type of study** | **Sugar used** | **Timescale and dosage** | **Overview of methods** | **Outcome measures** | **Key results and conclusions** |
| --- | --- | --- | --- | --- | --- | --- | --- | --- | --- | --- |
| 28 | | Hayashi et al., 2010^)^ | Japan | Borderline diabetes (fasting blood glucose 100-126mg/dl) (n=15) or healthy (n=11) volunteers  Age 22-69yrs | Randomized placebo-controlled, double-blind parallel-group study | PSI | Meal-loading study:  5g, with meal | Meal-loading study: 5g PSI given with meal, blood taken at 30min intervals for 120 min. | Meal-loading study: plasma glucose, AUC glucose for meal. | Meal-loading study: Plasma glucose was significantly lower at 30 and 60 min with all subjects after PSI meal compared to control meal.  AUC for glucose (mg/ml/dl) for test meal 578.8+/-2509.9, for control meal 6482.1+/-2953.8 (p<0.01 for difference)  Plasma glucose and AUC glucose significantly reduced with PSI, overall and in subjects with borderline diabetes but not in subgroup of healthy subjects. |
|  |  | |  |  |  |  | Safety study: 5g, 3x daily for 12 weeks | Long-term safety study: 5g with meals for 12 weeks. Fasting urine & blood taken at 2, 4, 8 and 12 weeks. | Long-term safety study: reported adverse effects. | Long-term safety study: No significant differences observed in nutritional intake. No persistent or serious adverse effects. |
| 29 | | Noronha et al., 2018 | Canada | Subjects (n=24) 12m, 12f with T2D (controlled with diet or OHAs, not insulin) Age 66+/-1.2yrs  BMI 27+/-0.9kg/m^2^ | Randomized controlled, double-blind crossover acute feeding equivalence | PSI | 0, 5 or 10g in 75g glucose solution | 75g OGTTs with 0, 5 or 10g fructose or PSI added (6 visits 1 week apart). Blood taken every 30 minutes for 120 minutes, plasma glucose and insulin measured. | iAUC for plasma glucose, iAUC for plasma insulin, absolute maximum concentrations (C_max_) for glucose and insulin. | With 10g PSI, significantly reduced iAUC for glucose (mol*min/l):  5g PSI -48.1 (SE24.7, p=0.051)  10g PSI -601.1 (SE24.7, p=0.015)  Significant linear dose response gradient for reduction. Significantly reduced absolute mean plasma glucose with 5g.  Equivalence test shows results within 20% equivalence boundaries, and reductions in glycaemic response were modest compared to oral antihyperglycaemic agents e.g. acarbose. |
| 30 | | Matsuo and Lu, 2011  ABSTRACT ONLY | Japan | Healthy subjects, (n=44) 15m, 29f | no details available | PSI | 6g, single dose before meal | 6g PSI or fructose given with normal lunch. Blood taken regularly for 120 minutes after meal. | Plasma glucose and insulin. | Significantly lower glycaemic response after PSI compared with fructose. |
| 31 | | Yamada et al., 2018  ABSTRACT ONLY | Japan | Healthy subjects (three trials, n=6, n=14, n=10) | Randomized, controlled single-blind crossover | RSS | 0, 30 or 50% of sucrose in test food/drink, single dose | Half of sucrose in drink (n=6) or food (n=14) replaced with RSS, or 0, 30 or 50% sucrose replaced with RSS in test drink (n=10). Blood collected at 5 time-points before and after ingestion. | iAUC for plasma glucose and insulin. | All foods and drinks containing RSS showed significantly reduced iAUC for glucose compared to sucrose control. No significant changes in iAUC for insulin were observed. |
| 33 | | Kwak et al., 2013 | Korea | Healthy (n=52) and hyper-glycaemic (n=33) Korean subjects | Randomised controlled, double-blind crossover | TAG | 5g, single dose before meal | 5g or 10g TAG or placebo (sucralose or erythritol) consumed before standard meal (356kcal, of which 59.57% - 53g - was CHO). Blood taken every 30 mins for 120 mins | Plasma glucose and insulin. | In subjects with hyperglycaemia, TAG significantly reduced AUC for glucose (4% decrease) and plasma glucose at 120 mins compared to placebo. With high dose TAG in healthy subjects there was a non-significant reduction in blood glucose, and significantly lower AUC for insulin and c-peptide. |
| 34 | | Nakamura et al., 2017  ABSTRACT ONLY | Japan | Healthy subjects (n=10, n=12) | Randomized placebo-controlled, single blind crossover | RSS | 0-35g single dose | 50g tolerance test: 50g sucrose replaced with RSS in ratio 0:10, 3:7, 5:5, 7:3. 10g tolerance test: 10g sucrose or 5g RSS with 5g sucrose. Blood glucose and insulin measured. | Blood glucose and insulin. AUC for glucose and insulin. | Significant reductions in AUC for glucose were seen with RSS:sucrose in ratios of 5:5 and 7:3. Significant reductions in AUC for insulin were seen with ratios of 3:7, 5:5 and 7:3. |
| 35 | | Tanaka et al., 2020  ABSTRACT ONLY | Japan | Young, healthy Japanese women | Single-blind, randomised crossover | PSI | 0, 1.8, 3.6 or 12.5g single dose in 50g chocolate | Blood taken before consumption of chocolate and at 1, 2, 4 and 6hours afterwards. | Free fatty acids, blood glucose and insulin and GLP-1 measured. | Post-prandial free fatty acids were increased and glucose and insulin decreased after consuming chocolate containing PSI compared to placebo. Enhanced GLP-1 secretion observed after PSI intake. |
| 36 | | Han et al., 2018 | Korea | Overweight Asian subjects (n=144)  Age 20-40yrs  BMI >/= 23 | Randomised double-blind placebo-controlled parallel study | PSI | 4g or 7g, 2x daily for 12 weeks. | Placebo (sucralose) or PSI given 2x daily after meals. Anthropometric measurements and blood samples taken every 4 weeks. | BMI, body composition, fasting blood glucose, HbA1c and lipids. | With PSI: significantly lower body fat percentage, body fat mass and BMI. Significantly lower total fat area, particularly subcutaneous fat, in high-dose PSI group compared to placebo.  No significant differences observed in plasma lipids, fasting blood glucose, HbA1c or leptin. |
| 37 | | Donner et al., 2010 | USA | Subjects (n=8, 4m, 4f) with T2D- poor glycaemic control.  Age 50.7 +/- 10.9yrs  BMI 36.7 +/- 5.1kg/m^2^ | Pilot intervention study, no control, no blinding. | TAG | 15g, 3x daily for 14 months. | TAG taken with regular, non-standardised meals. Body weight and vital signs recorded and blood taken every 2 months. Subjects questioned about adverse effects. | Body weight, plasma glucose, insulin, glycated haemoglobin, lipids. | Significant weight loss compared to baseline after 12 months (p=0.01).  Significant overall decrease in glycated haemoglobin, but this was non-significant when two patients who had started or increased medication were excluded.  Significant increases in HDL-cholesterol, no changes in plasma triglycerides, total cholesterol or LDL-cholesterol. All subjects experienced transient mild GI symptoms in the first two weeks, these were persistent in one subject. |
| 38 | | Ensor et al., 2014 | USA and India | Asian subjects (n=161) with T2D controlled with diet and exercise only. Majority male, around 50 yrs. | Prospective randomised parallel dose-ranging trial, single-blind (subjects blind to dosage) | TAG | 2.5, 5 or 7.5g 3x daily for 6 months. | After 8-week stabilisation period subjects randomised into three groups and given TAG 3x daily for 6 months. Medical examination at start and end of trial, regular blood tests. | Reduction in HbA_1c_ , fasting blood glucose and insulin, serum lipids, body weight. | Treatment success (defined as a 0.5 or greater decrease in %HbA1c) was greatest for 7.5g dose, but difference between doses was not statistically significant. Only 7.5g dose reduced fasting blood glucose after 6 months. Plasma triglyceride increased in 5.0g group, but no significant differences in total, LDL or HDL cholesterol.  Dose-dependent reduction in body weight observed. |
| 39 | | Hayashi et al., 2014^)^ | Japan | Healthy volunteers (n=34)  Age 42 +/- 2.7yrs  BMI 25.5 +/- 0.6kg/m^2^ | Randomised placebo-controlled, double-blind parallel-group study | RSS (6% PSI) | 30g RSS or 28g HFCS (each 114 kcal) given daily for 12 weeks | Test (RSS) or control (HFCS) drink taken 30mins before breakfast each day. Body weight, body fat ratio, blood pressure measured and blood samples taken after 0, 2, 4, 8 and 12 weeks. | BMI, body fat ratio, hip and waist circumference, plasma glucose, insulin, HbA1c, lipids, leptin and other biochemical parameters. | With RSS, hip circumference was significantly reduced compared to baseline after 4 weeks. Body weight BMI, body fat ratio and waist circumference were significantly reduced compared to baseline from week 8, however differences between RSS and HFCS groups were non-significant.  Retinol-binding protein (highly expressed in visceral fat) significantly reduced in RSS group at week 12 compared to baseline, and leptin significantly increased in RSS group at week 12 compared to baseline. |
| 40 | | Ensor et al., 2015 | USA and India | Asian subjects (n=480) with T2D controlled with diet and exercise only. | Placebo-controlled, randomised, double-blind, parallel-group phase 3 clinical trial. | TAG | 15g, 3x daily for 12 months. | After 8-week stabilisation period subjects randomised into two groups and given TAG or placebo (Splenda) 3x daily before meals. Medical examination on initial visit, blood taken every 2 months. | Reduction in HbA_1c_ , fasting blood glucose and insulin, serum lipids, body weight. | Significantly greater reduction in HbA1c observed in TAG group compared to placebo. Significant difference was seen earlier in the subgroup with baseline HbA1c <7.5%. Effect of lowering HbA1c was more pronounced in US population versus Indian population.  No observed effect of TAG on changes in body weight or BMI compared to placebo.  TAG group showed better reductions in total cholesterol and LDL compared to placebo from 4 months. |
| 52 | | Wu et al., 2012 | Australia | Healthy subjects (n=10) 7m, 3f  Age 28.2+/-4yrs  BMI 25.5+/-1.5 | Randomized single-blind crossover | TAG | 40g TAG-isomalt mixture (TIM) (16g tag), single dose before CHO-based meal | Subjects given preload of 40g glucose, TIM, OMG (non-metabolizable SGLT1 substrate) or 60mg sucralose, 20 min before meal (potato, glucose and egg yolk labelled with 13C octanoic acid). Breath samples and blood taken and GI sensations recorded. | Plasma glucose, insulin, GLP-1 and GIP, gastric emptying. | Glucose preload increased blood glucose immediately and iAUC for glucose was significantly higher at 30 mins after the meal, but there were no significant differences in iAUC for glucose over 240 mins. iAUC for insulin was significantly higher with glucose preload over 240min. No significant differences were observed between TIM and sucralose in terms of blood glucose or insulin iAUC. Plasma GLP-1 was significantly higher with glucose, OMG and TIM preloads than with sucralose. Plasma GIP was significantly higher with glucose and OMG preloads than with sucralose or TIM. Gastric emptying was slower with OMG and TIM preloads than with sucralose preload. Fullness after meal was reduced with sucralose.  Nonnutrient substrates of SGLT1 or poorly absorbed sweeteners (TIM) could be used instead of protein or fat preloads to stimulate GLP-1 and slow gastric emptying, therefore reduce post-prandial elevation in blood glucose in people with T2D. |
| 53 | | Van Opstal et al., 2019 | Netherlands | Caucasian men, age 18-25yrs, BMI 20-23 | Double-blind crossover | PSI | 23g single dose in ‘milkshake’ containing 0.33g protein, 5g fat. | Resting-state functional MRI carried out immediately before and for 15mins after consumption of shake containing glucose, fructose, sucralose or PSI (matched for sweetness). Visual analogue scores used to measure hunger and fullness. | Changes in blood oxygen level dependent signal, functional network connectivity and voxel-based connectivity in brain. | No significant differences in visual analogue scores between sweeteners.  Glucose and fructose decreased blood oxygen level dependent signal, and glucose increased eigen vector centrality throughout brain but decreased eigen vector centrality in mid-brain.  PSI and sucralose had no effect on blood oxygen level dependent signal, but sucralose increased eigen vector centrality in some brain regions.  The brain reward and satiety responses to low-calorie sweeteners was minimal compared to glucose and fructose. |
| 56 | | Braunstein et al., 2018 | Canada | Healthy volunteers (n=25, 13m,  12f)  Age 37+/-16yrs  BMI 24.7+/-3.4kg/m^2^ | Randomized controlled, double-blind, crossover acute feeding equivalence | PSI | 0, 5 and 10g added to 75g glucose in 500ml water. | OGTTs with fructose or PSI (6 visits 1 week apart). | iAUC for plasma glucose, iAUC for plasma insulin, absolute maximum concentrations (C_max_) of glucose and insulin. | iAUC for glucose (mmol/L*min) was reduced but effect was not significant.  5g PSI: -35 +/-22 (p=0.11).  10g PSI: -23 +/-22 (p=0.30)  Pooled PSI: -29=+/-16 (p=0.07)  No significant effect of PSI on iAUC for insulin, or C_max_ for glucose or insulin. Direction and magnitude of lowering of glycaemic response was similar to previous studies, but not significant because of high within-subject variability. |
| 57 | | Kimura et al., 2017 | Japan | Healthy volunteers (n=13, m5, f8)  Age 35.7 +/-2yrs  BMI 20.9 +/-0.7 kg/m^2^ | Randomized crossover, single blind | PSI | 5g, single dose before meal | 5g PSI or 10mg aspartame given 30 min before standard meal after 12h fast. Energy metabolism and blood biochemical parameters measured. | REE, CEE and FEE, RQ.  Plasma glucose, insulin and lipids. | No significant differences in REE. AUC for FEE increased and CEE reduced with PSI compared to control. With PSI, plasma glucose significantly reduced at 90 minutes, plasma free fatty acids significantly increased from 180 minutes. |
| 66 | | Iida et al., 2008  ABSTRACT ONLY | Japan | Healthy Asian subjects (n=20) 11m, 9f Age 20-39yrs | Randomised single-blind crossover | PSI | 0, 2.5, 5 or 7.5g single dose in solution with 75g maltodextrin | Subjects visited at intervals of >1week. Blood sampled before consumption of PSI/maltodextrin and at 30, 60, 90 and 120mins after. | Blood glucose, insulin. | PSI dose-dependently reduced the elevation of blood glucose and insulin after maltodextrin load, with significant effects at doses of 5g or greater.  7.5g PSI administered alone did not affect blood glucose or insulin. |
| 75 | | Han et al., 2018 | Korea | Healthy Asian subjects (n=30) Age 21-30yrs BMI 18.5-23 | Placebo-controlled tolerance testing | PSI | 0.1g – 0.6g daily, or 0.2g-1g per kg body weight, 2-5 times daily, increasing over 1 week. | Gradually increasing single daily dose over 6 weeks with 1-week washout to find max single dose for occasional consumption, or gradually increasing daily dose and frequency over 1 week to find max daily dose for regular ingestion. | Max single dose for occasional ingestion, max daily dose for regular consumption. | Maximum single dose with no reports of severe GI symptoms was 0.4g per kg body weight.  Maximum total daily intake for regular consumption with no reports of severe GI symptoms was 0.9g per kg body weight per day. |
| 89 | | Little et al., 2010 | UK | Healthy subjects (n=31) 16m, 15f, age 32.5 (SEM4.2), BMI 23.2 (SEM 0.7) | Single-blind, randomised. | TAG | 22.5 or 45g in 500ml water, single dose | Hexose sugars (glucose, galactose, fructose or TAG) at various osmolalities given alongside 13C-labelled sodium acetate, breath samples taken before ingestion and at 5min intervals for 45mins. | Gastric emptying (13C:12C ratio in breath samples) | At 22.5g dose (250mOsmol) TAG slowed gastric emptying, with no difference between glucose, fructose and water. At 45g dose (500mOsmol), glucose, fructose and TAG all slowed gastric emptying compared to water, with no differences between different hexoses. This effect was reduced when a CCK1 inhibitor was administered.  Gut-brain signalling affecting gastric emptying involves hexose-specific effects, independent of osmolality. TAG seems to slow gastric emptying more than other hexoses at physiological osmolalities. |

All reported differences are significant unless stated otherwise. PSI: D-psicose, TAG: D-tagatose, RSS: rare sugar syrup, HFCS: high-fructose corn syrup, TIM: TAG-isomalt mixture, OMG: O-methylglucose, T2D: type 2 diabetes, OHAs: oral hypoglycaemic agents, OGTT: oral glucose tolerance test, AUC: area under curve, BMI: body mass index, CHO: carbohydrate, HDL: high density lipoprotein, LDL: low density lipoprotein, HbA1c: glycated haemoglobin, GI: gastrointestinal, REE: resting energy expenditure, CEE: CHO energy expenditure, FEE: fat energy expenditure, RQ: respiratory quotient, GLP-1: glucagon-like peptide-1, GIP: gastric inhibitory polypeptide, SGLT1: sodium-glucose cotransporter 1.
